# Patients’ perspectives of eosinophilic granulomatosis with polyangiitis within the context of a clinical trial

**DOI:** 10.1016/j.ero.2025.08.009

**Published:** 2025-10-24

**Authors:** Peter A. Merkel, Bernhard Hellmich, Christian Pagnoux, Ulrich Specks, Michael E. Wechsler, David Badenoch, Alisa Hartsell, Vivian H. Shih, Lena Börjesson Sjö, Sofia Necander, Caroline Roberts, Jennifer Hanlon, Jennifer N. Hill, Eyra Perez, Julie Bailey, Calvin N. Ho

**Affiliations:** 1 Division of Rheumatology, Department of Medicine, University of Pennsylvania, Philadelphia, PA, USA; 2 Division of Epidemiology, Department of Biostatistics, Epidemiology, and Informatics, University of Pennsylvania, Philadelphia, PA, USA; 3 Klinik für Innere Medizin, Rheumatologie, Pneumologie, Nephrologie und Diabetologie, Medius Kliniken, Akademisches Lehrkrankenhaus der Universität Tübingen, Kirchheim unter Teck, Germany; 4 Mount Sinai Hospital, University Health Network, Toronto, ON, Canada; 5 Canadian Vasculitis Research Network (CanVasc), Toronto, ON, Canada; 6 Division of Pulmonary and Critical Care Medicine, College of Medicine and Science, Mayo Clinic, Rochester, MN, USA; 7 Department of Medicine, National Jewish Health, Denver, CO, USA; 8 Patient Research Partner, Vasculitis Patient-Powered Research Network, Philadelphia, PA, USA; 9 Late-Stage Respiratory and Immunology, BioPharmaceuticals Research & Development, AstraZeneca, Gaithersburg, MD, USA; 10 Late-Stage Respiratory and Immunology, BioPharmaceuticals Research & Development, AstraZeneca, Gothenburg, Sweden; 11 Patient Centered Solutions, IQVIA, New York, NY, USA; 12 Patient Centered Solutions, IQVIA, Wakefield, MA, USA; 13 Patient Centered Solutions, IQVIA, Mexico City, Mexico

## Abstract

**Objectives:**

Eosinophilic granulomatosis with polyangiitis (EGPA) is a complex, multisystem form of vasculitis. MANDARA (NCT04157348) was a phase 3 clinical trial comparing the efficacy and safety of benralizumab versus mepolizumab, in addition to standard of care, for relapsing/refractory EGPA. This qualitative interview substudy explored study participants’ experiences with EGPA and their participation in the trial.

**Methods:**

Thirty-eight patients from 5 countries participating in MANDARA opted into a longitudinal qualitative substudy, which comprised two 60-minute, semistructured, one-to-one telephone interviews conducted by trained interviewers between December 2019 and June 2023. Topics discussed included motivation to join the trial, time since diagnosis, symptoms, and impacts of EGPA and their degree of ‘bothersomeness’ and ‘disturbance’.

**Results:**

The desire to stop or reduce the use of oral glucocorticoids was the most frequently mentioned motivation for, and expectation of, participation in the trial. Comparison of interviews between the 2 time points suggested patients experienced reductions in the number and impact of EGPA-related symptoms and bothersomeness and/or disturbance ratings. Most improved symptoms included difficulty breathing, nasal congestion/discharge, and fatigue, and most improved impacts included ability to exercise, quality/quantity of sleep, and ability to engage in social activities.

**Conclusions:**

This study provides insights regarding patients’ perspectives of EGPA and their perceptions and experiences associated with receiving an anti-interleukin-5/receptor therapy. These findings highlight the need for effective treatments that allow patients to reduce their use of oral glucocorticoids and the importance of assessing patient experiences when gauging treatment efficacy in EGPA.

**WHAT IS ALREADY KNOWN ON THIS TOPIC**

• The perceptions and priorities of patients with eosinophilic granulomatosis with polyangiitis (EGPA) regarding their disease state differs from those of their clinicians, with patients reporting fatigue and energy loss as an important aspect of disease, whereas clinicians focus more on physical manifestations.

• The MANDARA phase III trial showed that benralizumab was non-inferior to mepolizumab in achieving remission in patients with EGPA.

**WHAT THIS STUDY ADDS**

• This study used protocol-defined qualitative methods to assess patients' perspectives of the symptoms and impacts of EGPA, with a focus on experiences before and after treatment within a clinical trial.

• Patients expressed a strong desire to stop or reduce the use of oral glucocorticoids, which was the most frequently mentioned motivation for participation in the trial.

**HOW THIS STUDY MIGHT AFFECT RESEARCH, PRACTICE, OR POLICY**

• These data provide insight into the perspectives of patients with EGPA receiving anti-IL-5/R therapies and highlight the need to prioritize treatments that allow patients to reduce oral glucocorticoid use.

• This study confirms the importance of capturing patient experiences when assessing treatment outcomes and efficacy in EGPA.

Alt-text: Unlabelled box

## INTRODUCTION

Eosinophilic granulomatosis with polyangiitis (EGPA) (Churg–Strauss) is a rare form of vasculitis characterised by asthma, sinusitis, pulmonary infiltrates, eosinophilia, and other manifestations that can be potentially organ- or life-threatening, including through involvement of the heart, lungs, skin, gastrointestinal tract, kidneys, and nervous system [[1], [2], [3], [4]]. Although the clinical presentation of EGPA is highly variable [2,5], the disease generally has a major impact on patients’ health-related quality of life (HRQoL). For example, patients often note that once they had EGPA, they were less able to work or perform daily activities [6]. In addition to the symptoms and impacts of the disease itself, extensive exposure to oral glucocorticoids (GCs) and high healthcare resource utilisation (eg, numerous visits to specialist physicians) can negatively impact a patient’s daily life and well-being.

Clinical trials in vasculitis frequently focus on clinician-reported outcomes of disease activity [7] and damage [8,9]. Physician experts in vasculitis, regardless of their specialty, broadly agree on the disease- and treatment-related items of activity and damage important to measure in a clinical trial [9,10]. For example, the Birmingham Vasculitis Activity Score [7] captures a broad spectrum of active clinical manifestations of vasculitis, while damage, assessed using the Vasculitis Damage Index [8], is often used as a proxy for the chronic burden of disease. However, symptoms and HRQoL impacts can only be reported reliably by patients. Previous research showed that patients’ perceptions of rheumatic diseases differ from those of clinicians [9,[11], [12], [13], [14]]. In 1 survey of 264 patients with vasculitis, the majority rated fatigue or energy loss as the most important aspect of the disease in their daily life; pain, musculoskeletal symptoms, and the financial impact of the disease were also commonly mentioned [12]. By contrast, clinicians tend to focus on physical manifestations rather than fatigue and psychological or social impacts, which are not directly observable by a clinician and can only be known through patient reports [9,12,13]. Additionally, symptoms may vary day to day, and symptoms reported during a clinic visit may not provide a full overview of the patient’s experience due to challenges with recall, time limitations, or a reluctance to report some problems [15]. Greater insight into the experiences of patients with EGPA and how these experiences change with treatment would support shared treatment decision-making and guide outcome assessment in clinical trials.

Clinical trials testing the efficacy of treatments for EGPA have advanced the care for this disease [16,17], and new treatments may also offer considerable benefits to patients’ HRQoL [18]. MANDARA (NCT04157348) was a randomised, double-blind, multicentre, phase 3 clinical trial comparing the efficacy and safety of 2 injectable biologics (benralizumab and mepolizumab) in patients with relapsing or refractory EGPA [17]. The trial showed that benralizumab was noninferior to mepolizumab with regard to achieving remission. A qualitative interview substudy was conducted as part of the trial to characterise patients’ experiences with symptoms and impacts associated with EGPA and their motivations for and experiences of participating in a trial; the results of this substudy are reported here.

## METHODS

In the MANDARA trial, eligible adults (≥18 years) were randomly assigned in a 1:1 ratio to receive benralizumab (30 mg, as 1 injection) or mepolizumab (300 mg, as three 100 mg injections) subcutaneously every 4 weeks for 52 weeks; patients with organ- or life-threatening EGPA within 3 months were excluded from participation. The full methodology and results of the trial have been published [17].

### Substudy design and participant selection

Participants who met the criteria of residence in the United States, the United Kingdom, Canada, Germany, or Belgium, with proficiency in English (for United States, United Kingdom, or Canada), French (Canada or Belgium) or German (Germany) were given the opportunity to opt in to this longitudinal, qualitative substudy exploring the concepts relevant and important to patients and their disease-related experiences throughout the trial. Interested participants provided consent to participate via an addendum to the clinical trial informed consent form; all procedures were performed in compliance with the relevant laws in each country and the substudy was approved by the institutional review boards for the relevant sites. Additional details on researchers (including a COnsolidated criteria for REporting Qualitative research [ COREQ] checklist [19]) and translation methodology are available in the Supplementary Methods.

### Interview process and outcomes

The qualitative substudy comprised two 60-minute, 1:1 semistructured telephone interviews conducted by trained moderators at 2 time points between December 2019 and June 2023 (Supplementary Methods and Supplementary Fig S1). Interview 1 was completed at least 7 days and up to 21 days after initiation of treatment, whereas interview 2 was completed at least 7 days and up to 21 days after the final dose of blinded treatment (week 48). At the time of the interviews, the treatment arm to which participants had been randomised to was blinded. Three discussion guides were developed to facilitate the interviews (1 each to conduct interviews 1 and 2, and 1 for participants who took part in interview 2 only). The discussion guides were designed to be semistructured, with open-ended questions to facilitate a free flow of discussion, rather than being strictly guided by the interviewer.

Topics discussed in interview 1 included participant demographics and characteristics; motivation to participate in the trial; expectations from participation in the trial; participants’ definitions of treatment success, symptoms, and impacts of EGPA on daily life; bothersomeness or disturbance ratings (scale from 0 to 10, where 0 was not bothersome at all and 10 was extremely bothersome) before joining the trial; and most bothersome symptoms. Impacts could refer to negative or positive changes in the patient’s day-to-day life because of symptoms of EGPA, including, but not limited to, physical and physiological impact of the disease, its symptoms and treatment; emotional and psychological effects of the disease, its management or prognosis; social impact; effect on relationships; impact on the patient’s ability to care for themselves and others; time and financial impact of the disease and its management; and considerations on the impact of EGPA on the patient’s family.

Topics discussed in interview 2 included symptoms and impacts experienced during study treatment (since interview 1) and bothersomeness or disturbance ratings at the time of interview 2; perceptions of success of the study treatment; and any changes in experience of symptoms and impacts since interview 1. Bothersomeness/disturbance ratings were averaged to calculate an average disturbance rating.

The most frequently patient-reported symptoms and impacts are reported as the proportion of participants who mentioned each concept out of the total number of participants who completed each interview. Participants could report more than 1 motivation, expectation, or definition of success, and for these, percentages were reported out of the total number of participants who completed interview 1. Participants’ verbatim quotes, representative of the critical findings, are also reported.

Additionally, 6-item Asthma Control Questionnaire (ACQ-6) scores, Short Form 36 Health Survey Questionnaire version 2 (SF-36v2) scores, and daily dose of oral GCs were extracted from the clinical trial data for the substudy participants; changes from baseline (initiation of treatment) are reported for each.

## RESULTS

### Substudy population

**Figure 1:**
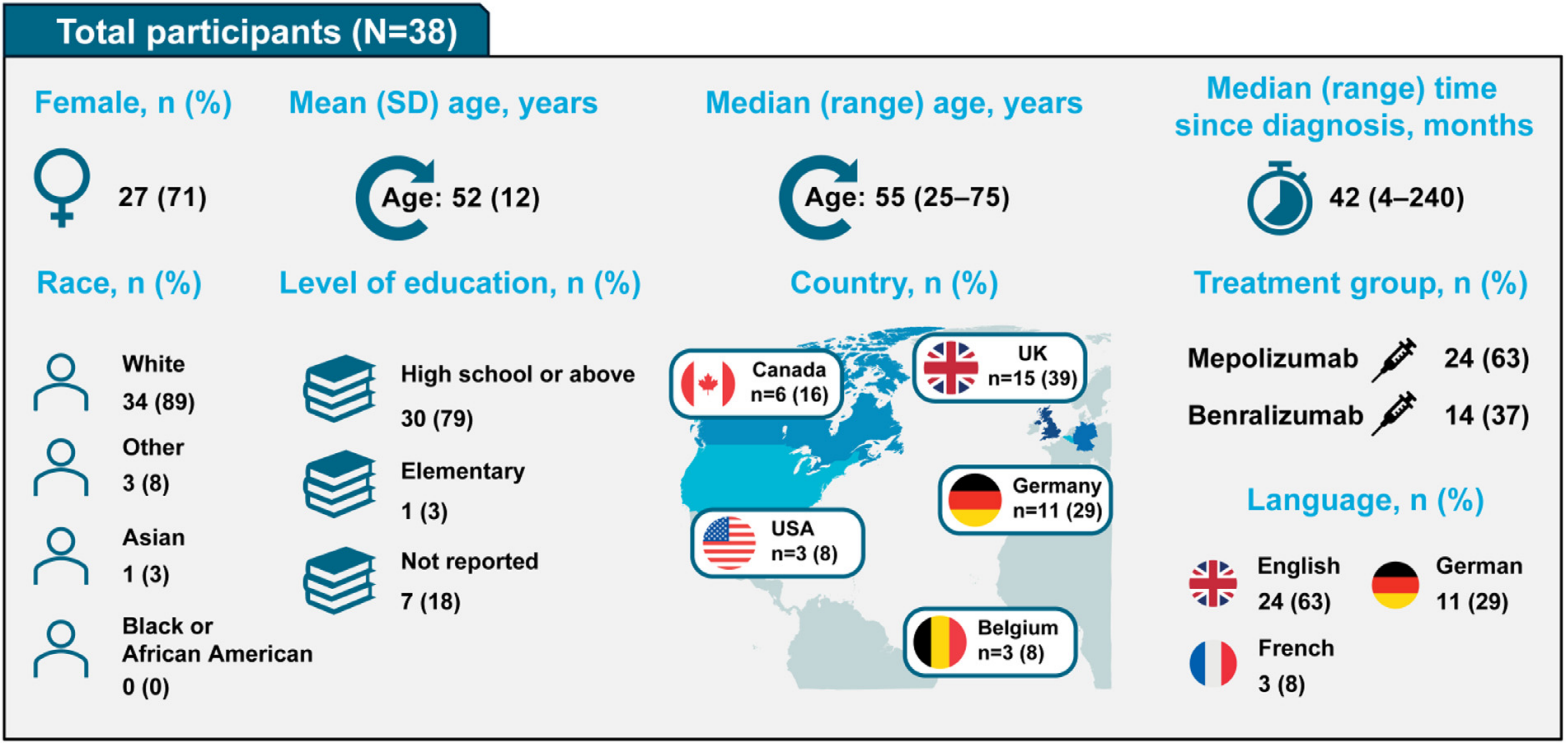
Demographics and baseline characteristics. Full demographics and baseline characteristics are reported in Supplementary Table S1. Map graphic created with MapChart. Race was asked as an open-ended question in recognition of the variability in self-categorisation across the countries in the study.

Thirty-eight patients consented to participate in the substudy: 35 (92%) participants completed interview 1; 32 (84%) completed interview 2, and 29 (76%) completed both interviews (Supplementary Fig S2). Median age was 55 (range: 25-75) years, and 27 were female (71%), with 29 (76%) from Europe, 6 (16%) from Canada, and 3 (8%) from the United States. Median time since diagnosis was 3.5 years (42 months, range of 4-240 months) (Fig 1, Supplementary Table S1).

ACQ-6 and SF-36v2 scores are reported in Supplementary Tables S2 and S3. Mean (SD) daily dose of oral GCs (prednisolone/prednisone equivalent) was 10.4 (4.4) mg at baseline (week 0) of the trial (Supplementary Table S4). Once the trial was unblinded, it was revealed that 14 (37%) of the interview substudy participants had been randomised to benralizumab and 24 (63%) to mepolizumab (Fig 1, Supplementary Table S1).

For consistency and ease of interpretation, numbers and proportions (n [%]) of participants are reported out of the total number of participants who completed the interview at each time point.

### Symptoms and impact of EGPA

Prior to joining the trial, participants experienced a wide range of symptoms affecting different organ systems. Their symptoms of EGPA also impacted their HRQoL in various ways. The symptoms and impacts mentioned in each interview are provided in full in Supplementary Tables S5-S8.

#### Interview 1: before joining the clinical trial

**Figure 2:**
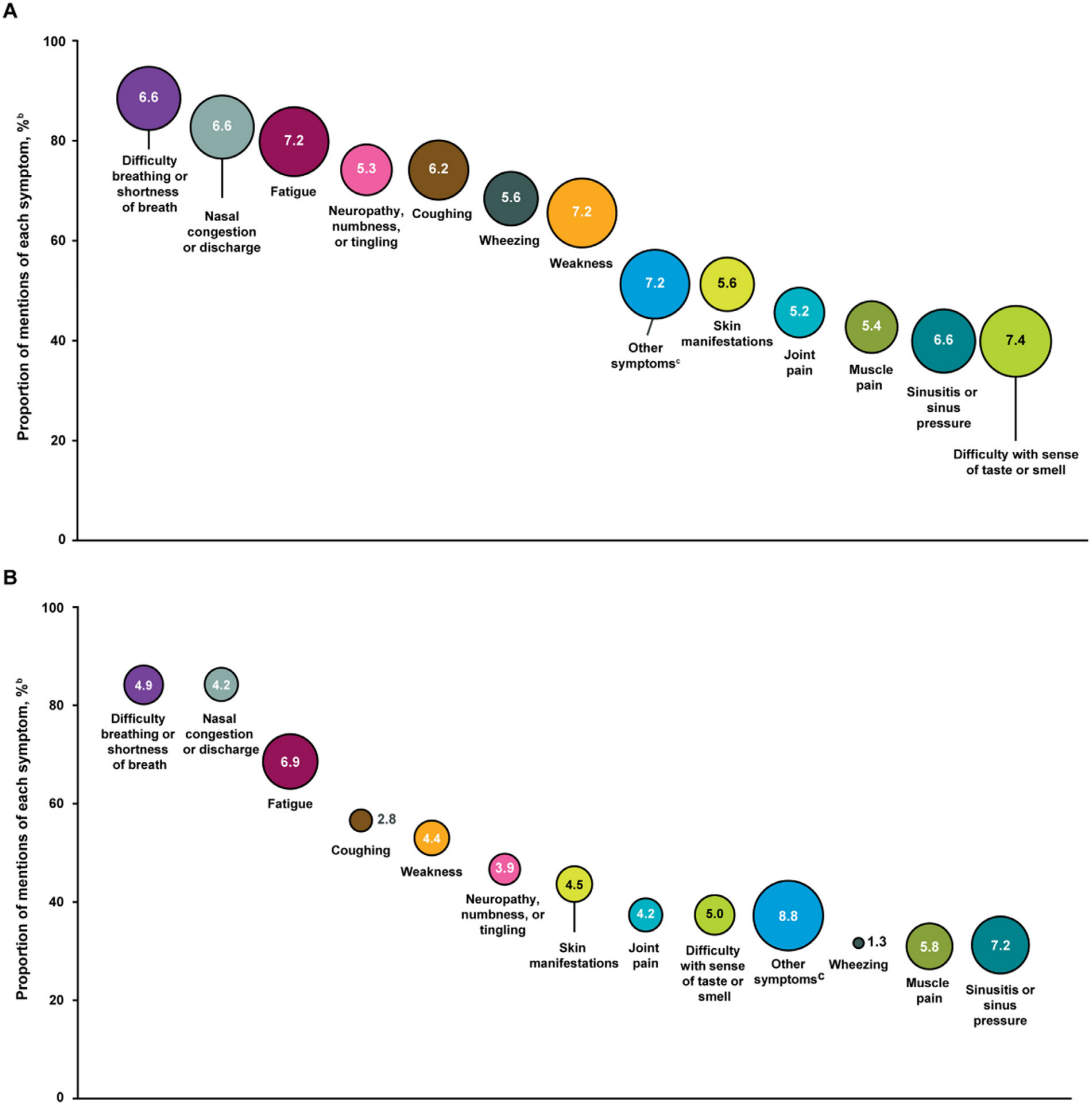
The most frequently patient-reported (A) symptoms and (B) impacts of eosinophilic granulomatosis with polyangiitis and related average disturbance ratings^a^ at (A) interview 1 (n = 35) and (B) interview 2 (n = 32). ^a^The size of the circles represents the average disturbance rating (0-10 scale, where 0 is not bothersome at all and 10 is extremely bothersome). The number of participants who provided a bothersomeness/disturbance rating for each symptom or impact is reported in Supplementary Tables S5-S8. ^b^Proportion of the total participants who completed interview 1 (n = 35); 3 participants completed only the second interview. ^c^Other symptoms and impacts (those reported by ≤3 participants) are listed in the Supplementary Material.

**Symptoms**. At interview 1, participants reported 42 symptoms and other related effects about their experience of EGPA before joining the clinical trial. The most frequently reported symptoms included difficulty breathing or shortness of breath; nasal congestion or discharge; fatigue; neuropathy, numbness, or tingling; coughing; wheezing; and weakness (Fig 2A, Supplementary Table S5).

**Figure 3:**
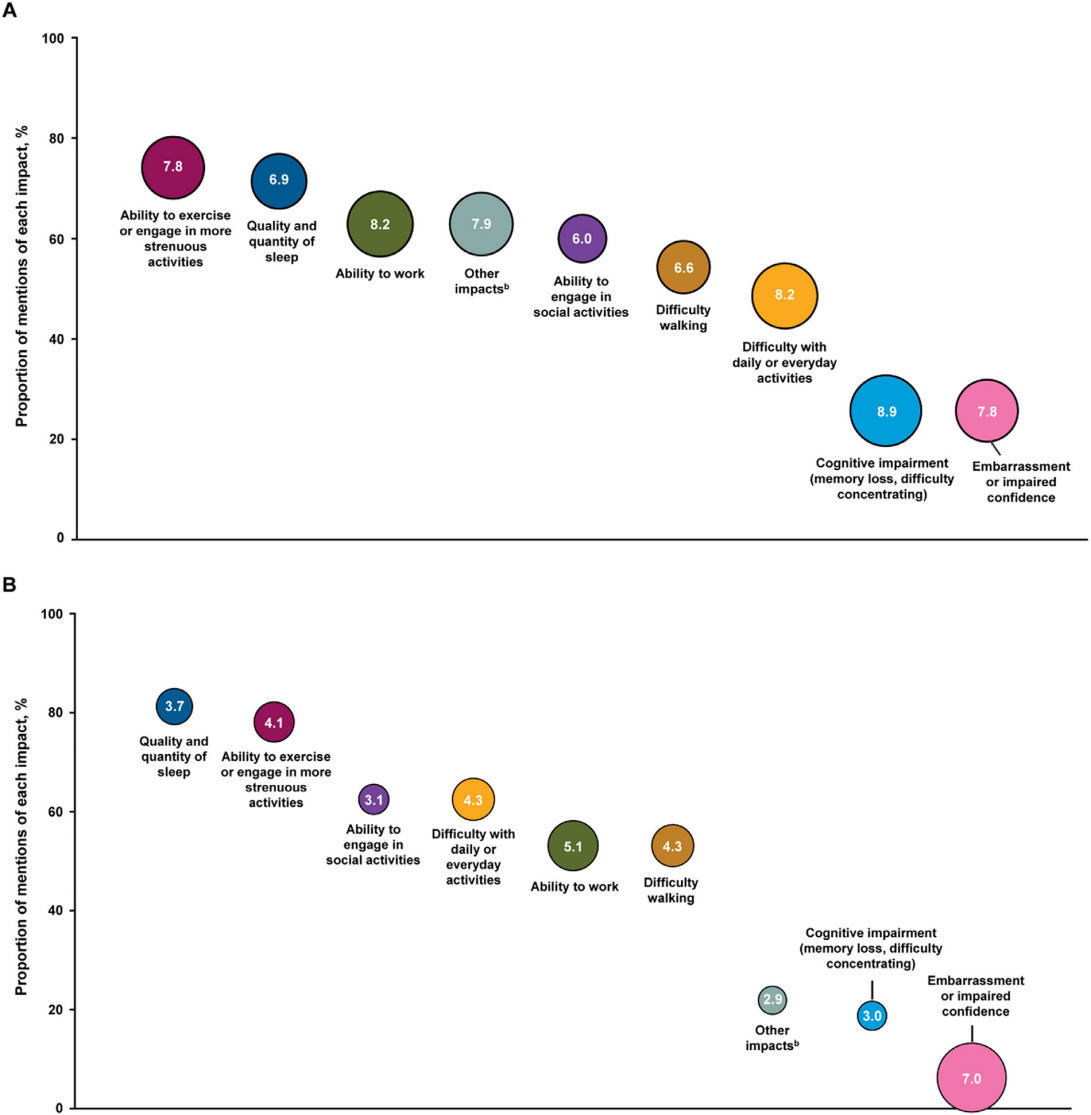
The most frequently patient-reported (A) symptoms and (B) impacts of eosinophilic granulomatosis with polyangiitis and related average disturbance ratings^a^ at (A) interview 1 (n = 35) and (B) interview 2 (n = 32). ^a^The size of the circles represents the average disturbance rating (0-10 scale, where 0 is not bothersome at all and 10 is extremely bothersome). The number of participants who provided a bothersomeness/disturbance rating for each symptom or impact is reported in Supplementary Tables S5-S8. ^b^Other symptoms and impacts (those reported by ≤3 participants) are listed in the Supplementary Material.

**Impact**. Participants reported experiencing 23 impacts related to their experience of EGPA before joining the clinical trial. The most frequently reported impacts included ability to exercise or engage in more strenuous activities; quality and quantity of sleep; ability to work; other impacts, ability to engage in social activities; difficulty walking; and difficulty with daily or everyday activities (Fig 3A, Supplementary Table S6).

#### Interview 2: during the clinical trial

**Figure 4:**
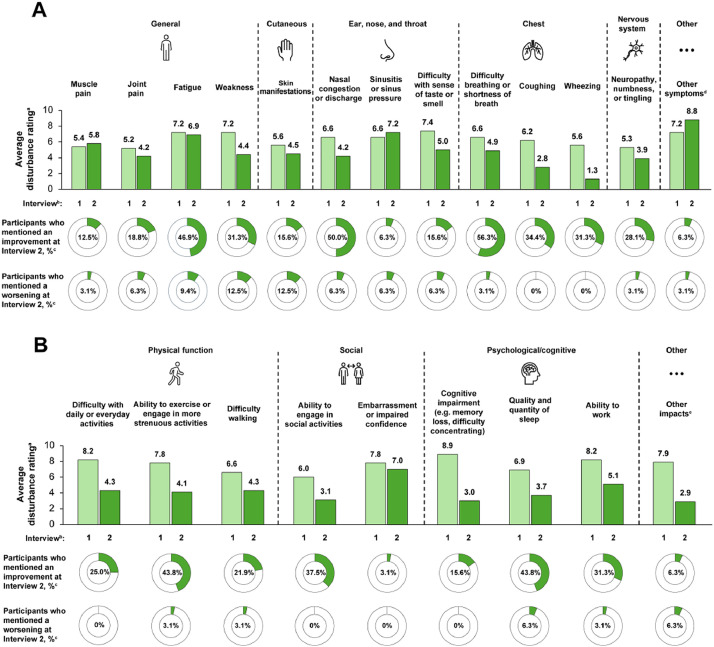
Average disturbance ratings^a^ for (A) symptoms and (B) impacts of eosinophilic granulomatosis with polyangiitis at interviews 1 and 2,^b^ and proportion of participants who mentioned an improvement or worsening at interview 2.^c a^Average disturbance ratings (0-10 scale, where 0 is not bothersome at all and 10 is extremely bothersome) were based on the number of participants who provided a quantitative rating (reported in Supplementary Tables S5-S8), which was not always the same as the number of participants who endorsed the symptom. Some participants provided qualitative descriptions and, despite gentle encouragement from the interviewer, did not provide a quantitative number. ^b^Six participants completed only the first interview; 3 participants completed only the second interview. ^c^Proportion of the total participants who completed interview 2 (n = 32). Participants may have experienced improvements or worsening since interview 1, but did not specifically mention them during interview 2. ^d^Other symptoms (those reported by ≤3 participants) are listed in the Supplementary Material. ^e^Other impacts (those reported by ≤3 participants) are listed in the Supplementary Material.

**Symptoms**. Participants reported experiencing 39 symptoms and other related effects concerning their experience of EGPA at interview 2. The most frequently reported symptoms included difficulty breathing or shortness of breath; nasal congestion or discharge; fatigue; coughing; weakness; neuropathy, numbness, or tingling; and skin manifestations (Fig 2B, Supplementary Table S7). Wheezing and coughing showed the greatest changes in average disturbance rating for bothersomeness/disturbance (all reductions) from interview 1 (Fig 4A, Supplementary Tables S5 and S7).

**Impact**. Participants reported experiencing 22 impacts related to their experience of EGPA at interview 2. The most frequently reported impacts included quality and quantity of sleep; ability to exercise or engage in more strenuous activities; ability to engage in social activities; difficulty with daily or everyday activities; ability to work; and difficulty walking (Fig 3B, Supplementary Table S8). Average disturbance ratings for bothersomeness/disturbance decreased across all domains from interviews 1 to 2 (Fig 4B, Supplementary Tables S6 and S8).

#### Participants’ perceptions of the association between symptoms and impacts

Quotes representing the patient experience of the data reported here are included in Supplementary Table S9. In interview 1, sleep disturbance was reported in 25/35 (71.4%) participants and included difficulty falling asleep and waking during the night, which, in some cases, impacted their ability to perform daily activities (eg, doing laundry, taking a shower), for example, ‘I don’t sleep well. I go to bed, and I tend to wake up several times in the night for various reasons, sometimes for no reason at all. I just wake up’. Sleep disturbance was attributed to nasal congestion and/or difficulty breathing in 9/35 (26%), oral GC use in 12/35 (34%), and coughing in 3/35 (9%) participants. Other EGPA-related sleep disturbance symptoms in 5/35 (14%) participants were as follows: chest tightness, joint pain, diarrhoea, hot flashes, and night sweats.

Fatigue, tiredness, weakness, dizziness, and heart rhythm disturbances were reported by 5/35 (14%) participants in relation to their ability to exercise or engage in more strenuous activities, and sometimes led to avoidance of these activities to the detriment of their emotional well-being and how they engaged with their children. Fatigue or tiredness was reported in 8/35 (23%) participants in relation to difficulty engaging in social activities, such as having dinner with friends or family, eating out, or playing sports, for example, ‘Exhaustion was the number one from the beginning. This exhaustion, they can’t explain it. It’s just you get extremely tired out of nowhere. That was the biggest thing that impacted my life…the fatigue’. Other symptoms that impacted social activities, although less frequently reported, included difficulty with sense of taste, pain, hearing loss, and wheezing (1/35, 3%). Social lives impacted by EGPA were reported by 7/35 (20%) participants, for example, by the inability to dance, intolerance to alcohol, the burden associated with having to carefully plan when going out, or feeling afraid of spoiling an event, for example, ‘From, I think, when I had my really first flare-up, my social life has gone to zero because I’m just too tired’. Some described embarrassment related to having ‘moon face’, a side effect of the long-term use of oral GCs in which the face appears puffy or rounded, which may negatively impact the patient’s confidence and self-esteem, for example, ‘I had this proverbial moon face. Sometimes, I then didn’t want to see friends at all. I hid myself away’.

Difficulty walking was reported by 5/35 (14%) participants in relation to shortness of breath, weakness 10/35 (28%), joint pain 4/35 (11%), fatigue or tiredness 3/35 (9%), and muscle pain 2/35 (6%). This had an emotional impact on 3/35 (9%) participants and included feeling depressed or frustrated, or experiencing changes in their family dynamic, for example, ‘I also had some depression because I was no longer able to go jogging. That was something that was really important for me*’.*

Fatigue or tiredness, pain (in muscles, hands, and feet), hearing loss, dizziness, wheezing, nasal congestion, difficulty breathing, and impairment were reported by 5/35 (14%) participants as impacting on their ability to work, with mentions of feeling forced to leave the workforce earlier than the customary retirement age in their country due to tiredness, fatigue, or pain in the arms and legs (related to neuropathy). Psychological or emotional implications of not being able to work were reported by 4/35 (11%) participants, including stress, embarrassment, and/or feeling they were behind in their career due to symptoms of EGPA and/or related impact, for example, I feel like I can’t really cope in a job like management. It’s just too strenuous for me’.

Embarrassment or impaired confidence was the most frequently reported emotional or psychological concept at interview 1, by 9/35 (26%) participants, but this was more infrequently reported at interview 2, by 2/32 (6%) participants. Fear or worry was reported by 4/32 (11%) participants in interview 1, but this was more frequently reported at interview 2, by 11/32 (34%) participants. Hope for the future or positive outlook was reported by no participants at interview 1, but a more positive outlook became evident at interview 2 and was reported by 8/32 (25%) participants. Depression was reported by 8/35 (23%) at interview 1, but less frequently at interview 2, by 3/32 (9%) participants.

#### Changes in symptoms and impact of EGPA

Towards the end of the double-blind period, participants in the interview substudy were still experiencing symptoms of EGPA. However, for many participants, the symptoms and their related impacts had improved over the course of the trial. Quotes representing the participants’ experience of changes in symptoms and impact are included in Supplementary Table S10.

At interview 2, participants reported an improvement in 34 symptoms and worsening of 20 symptoms. The most frequently mentioned improved symptoms included difficulty breathing or shortness of breath; nasal congestion or discharge; fatigue; coughing; weakness; and wheezing. The most frequently mentioned worsened symptoms included weakness, skin manifestations, and fatigue (Fig 4A, Supplementary Table S10).

The most frequently mentioned improvements on impacts included quality and quantity of sleep; ability to exercise or engage in more strenuous activities; ability to engage in social activities; ability to work; difficulty with daily or everyday activities; difficulty walking; fear or worry; and cognitive impairment, such as memory loss or difficulty concentrating. The most frequently mentioned worsening of impacts included quality and quantity of sleep and other impacts (Fig 4B, Supplementary Table S10).

### Asthma control and HRQoL questionnaires

On average, the participants of the substudy experienced improvements in their asthma symptoms, with a mean (SD) change from baseline score of −0.69 (0.93). Thirty-eight participants also experienced clinically meaningful improvements in various aspects of HRQoL as measured by SF-36v2; mean changes from baseline at week 52 in the mental component summary, physical functioning, role limitations due to physical health, and role limitations due to emotional problems domains exceeded prespecified group-level responder definitions (Supplementary Tables S2 and S3).

### Motivations for participation in the clinical trial, expectations, and definition of treatment success

Reduction or elimination of the use of oral GCs was the major reason why participants joined the MANDARA trial. Three motivations relating to the avoidance of oral GCs were reported by 12/35 (34%) participants in interview 1: 11/35 (31%) wanting to stop using oral GCs; 7/35 (20%) wanting to avoid the long-term side effects of oral GCs, and 6/35 (17%) wanting to reduce the use of oral GCs. Additionally, 9/35 (26%) reported wanting to help other individuals with EGPA, 4/35 (11%) wanting to improve symptoms, 4/35 (11%) due to worsening symptoms, 1/35 (3%) wanting to receive a novel treatment, 1/35 (3%) being unable to afford prior treatment, and 1/35 (3%) due to recommendation.

Expectations for participation in the trial were described as the desire to stop or reduce the use of oral GCs and were reported by 14/35 (40%) participants, to improve symptoms or reduce damage by 9/35 (26%), feel better or increase sense of well-being by 9/35 (26%), have closer monitoring by 3/35 (9%), help other patients with EGPA by 1/35 (3%), and better understand the disease by 1/35 (3%).

**Figure 5:**
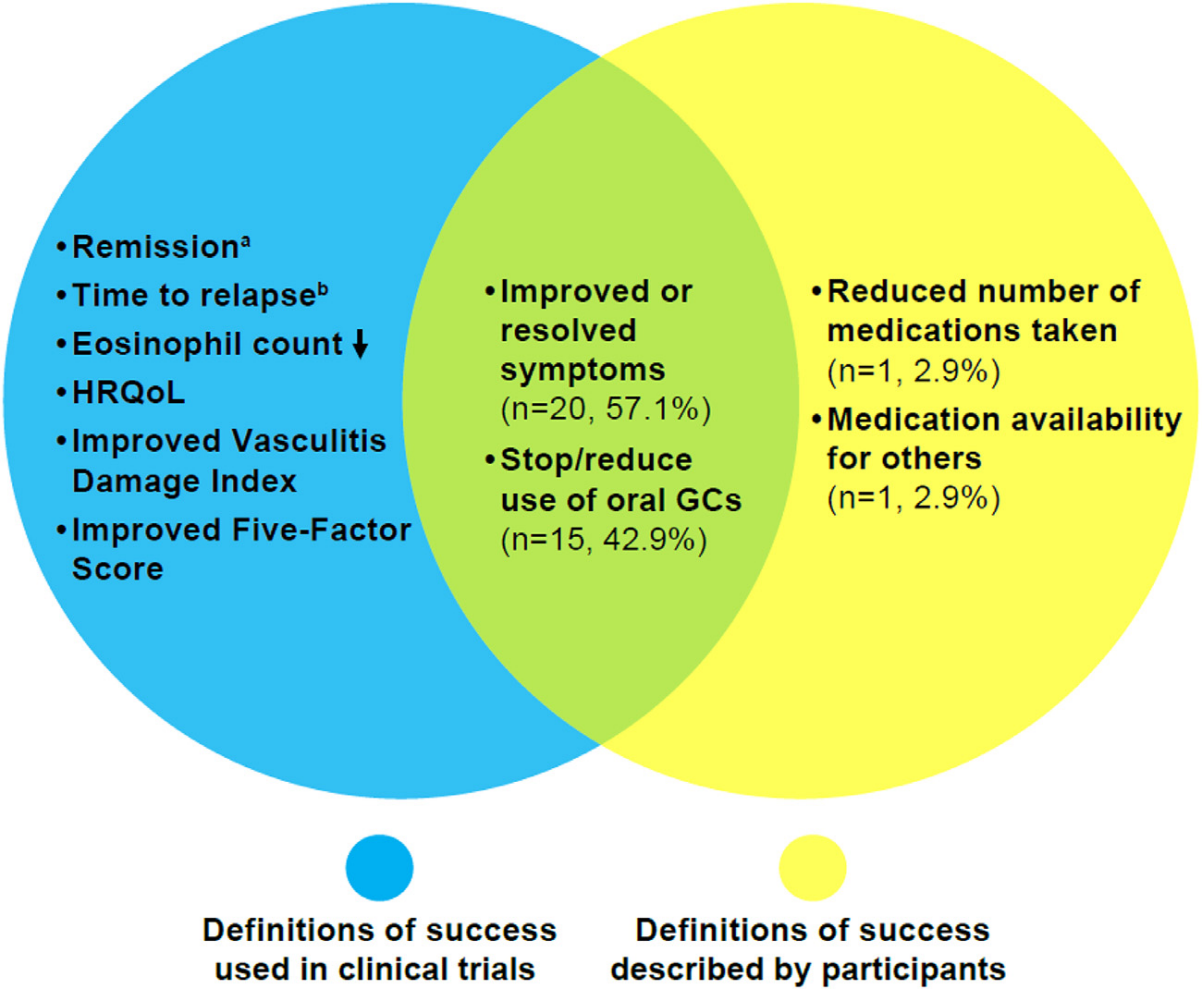
Comparison of definitions of treatment success as measured in clinical trials versus those described by the participants. Participants could give more than 1 definition of success. Percentages out of n = 35; 3 participants did not provide a response or only completed interview 2. GC, glucocorticoid; HRQoL, health-related quality of life. ^a^Birmingham Vasculitis Activity Score = 0 and prednisone (or equivalent) dose ≤7.5 mg/d. ^b^Recurrence of clinical signs or symptoms attributable to active disease following a period of remission or the need for an increase in dose of oral GCs or initiation of or increase in immunosuppressant use.

The most frequently reported definitions of treatment success included improving symptoms reported by 16/35 (46%) participants, reducing or stopping the use of oral GCs 15/35 (43%), and symptom resolution 4/35 (11%) (Fig 5).

### Perceptions of success of study treatment

Of the 32 participants who completed interview 2, 26 described their perceptions regarding the success of the study treatment. Twenty-three participants considered their study treatment to be successful, with 8 reporting the stopping or reduction of oral GC use as a reason for treatment success. Among the total 38 participants, the average daily dose of oral GCs (median, minimum–maximum) decreased from 10.0 (7.5-25.0) mg at baseline to 1.74 (0.0-10.0) mg during weeks 49 to 52 of the trial, amounting to an 87.5% reduction from baseline (Supplementary Table S4). Participants also alluded to improved HRQoL and reductions in side effects from oral GCs, such as weight stabilisation and improvements in symptoms such as breathing, energy, or ability to exercise.

## DISCUSSION

This substudy of the phase 3 MANDARA clinical trial [17] provides insight into the patient experience of EGPA and the perspectives and experiences of such patients before and during their participation in a clinical trial and informs research methods to study this disease.

Overall, comparison of the interviews with participants between the 2 time points suggested reductions in the number of EGPA-related symptoms and impacts and bothersomeness and/or disturbance ratings reported over the course of the trial. These findings parallel the observed improvements in symptoms of asthma and HRQoL assessed using patient-reported outcome questionnaires and improvements in disease activity seen in the trial population as measured by physician–investigators. Thus, patient-reported outcomes complement clinician-reported outcomes to provide a more comprehensive assessment of the impact of targeting [16] or depletion [20] of eosinophils in the treatment of EGPA.

The findings of this analysis are broadly consistent with other work on patients’ perspectives in antineutrophil cytoplasmic antibody-associated vasculitis, although a notably high proportion of participants reported that EGPA impacted their quality and quantity of sleep. The sleep disturbances in this study were attributed by patients to airway manifestations (nasal congestion, difficulty breathing, coughing), as well as the side effects from oral GCs [21]. Some participants in the current study transitioned to a more positive outlook between interviews 1 and 2, with fewer mentions of depression at interview 2. These are important findings; the emotional impact of EGPA on the mental health of patients is often overlooked during routine clinical visits and in clinical trials. It is important that patients’ mental health and emotional well-being are addressed both in clinical practice and trials.

Participants’ key motivations to join the MANDARA trial included (1) a desire to stop or reduce their use of oral GCs to avoid the long-term side effects of this therapy, although participants were not specifically asked about their use of oral GCs, and (2) a desire for improved control of EGPA-related symptoms. Participants described being ‘tired of taking steroids’, that it was their ‘biggest dream’ to stop or reduce their use of oral GCs, and that ‘steroids’ were the only drug that they had never wanted to take. Patients are generally aware of the risks of toxicity of oral GCs, and they are sometimes reluctant to pursue treatment if they or their loved ones have experienced the devastating side effects of oral GCs [22]. Clinicians aim to determine the minimally effective dose of GCs, and reducing the use of oral GCs is often the primary reason for initiating other immunomodulating therapy for EGPA.

Participants’ motivations also related to improving EGPA-related symptoms and impacts on quality of life, in particular difficulty breathing or shortness of breath, nasal congestion or discharge, fatigue, neuropathy, numbness, tingling, coughing, wheezing, and weakness. Comparison of interviews 1 and 2 suggested participants experienced reductions in difficulty breathing, nasal congestion/discharge, and fatigue and improved their ability to exercise, their quality/quantity of sleep, and their ability to engage in social activities.

These data confirm the importance and utility of including patients’ perspectives of illness when measuring the impact of treatment. Additionally, the results of this study should influence the choice of both outcome measures and the endpoints of interest for clinical trials in EGPA. Perhaps the clearest message from patients is to prioritise treatment strategies that mitigate against the many negative consequences of treatment with oral GCs.

There are several strengths to this study. This work offers insight into patients’ lived experiences of EGPA, specifically, and their views of participating in a clinical trial. The interviews were semistructured to allow free-flowing discussion between the participant and moderator. Data were collected from participants with broad geographical distribution, and the inclusion of patient research partners in the analysis and dissemination of these findings allowed for further refinement and insight from the patient perspective, which is of particular benefit to clinical research involving rare diseases [23]. The qualitative analysis of the interviews was complemented by quantitative data from the main clinical trial, allowing the results from both methodologies to be put into context.

There are also important limitations to this study to consider. Because the substudy was embedded in a larger interventional trial, with interview 1 taking place between 7 and 21 days after initiation of study treatment to allow for patient schedule flexibility, preclinical trial symptoms and impacts reported by participants may be subject to recall bias. Additionally, because the study treatment had already been administered at the time of interview 1, this may have impacted participants’ responses regarding their experiences with EGPA prior to joining the clinical trial. Participants may also perceive the severity of their symptoms or impact differently from one another, which likely affected the bothersomeness/disturbance ratings reported; as such, average disturbance ratings should be interpreted with caution.

Selection bias is another common limitation of qualitative interview studies. Patients who opted into participating in the trial and opted into the interview substudy may not be representative of the full study population. Additionally, information provided about the study design and objectives may have altered some patient perceptions, for example, by the high priority patients assigned to GC-related side effects in their experiences with EGPA. However, given that most patients who opted to join a trial experienced a lengthy patient journey, it is likely that they already had discussions with healthcare providers regarding these issues.

Similarly, the substudy population and the full study population may not be generalisable to the overall population with EGPA because of variation in age, sex, geography, and disease extent and history. The substudy population also differs from the full study population; in the substudy, there were 71% versus 60% females, and a longer time since diagnosis (3.5 vs 2.8 years; Supplementary Table S1). The reason for these differences is 2-fold. First, the substudy was only conducted in a few of the countries in the MANDARA trial. Second, recruitment for the substudy was on a voluntary basis. Patients who had more free time or had a desire to be more vocal about their disease experiences may have been more likely to opt into the substudy. Despite this, endpoint values were similar in the substudy population compared with the full study population, with only the daily dose of oral GC differing to any extent (Supplementary Table S11). Although the recruitment materials for the substudy were standardised and approved by local ethics committees, the discussions that patients had with site staff about study procedures could not be standardised and may have led to additional biases.

Finally, most of the interviews were conducted during the COVID-19 pandemic, which may have affected both the symptoms and impacts participants experienced and/or reported (eg, impacts on social life).

### Conclusions

This qualitative study provides important insights into patients’ perspectives of living with EGPA, and of their perceptions and experiences associated with receiving an anti-interleukin-5/receptor therapy and of participation in a clinical trial. These findings highlight the importance of patient-reported outcomes in the assessment of EGPA and the need for effective treatments that help patients reduce the use of oral GCs. These results also help guide treatment expectations and inform the choice of outcomes for future clinical trials.

## Competing interests

PAM reports receiving consulting fees from AbbVie, Alpine, Amgen, ArGenx, AstraZeneca, Boehringer Ingelheim, Bristol Myers Squibb, CSL Behring, GlaxoSmithKline, iCell, Interius, Kinevant, Kyverna, Metagenomia, Neutrolis, Novartis, NS Pharma, Q32, Quell, Regeneron, Sanofi, Sparrow, Takeda, and Vistera; research support from AbbVie, Amgen, AstraZeneca, Boehringer Ingelheim, Bristol Myers Squibb, Eicos, Electra, GlaxoSmithKline, Neutrolis, and Takeda; stock options from Kyverna, Q32, Lifordi, and Sparrow; and royalties from UpToDate. BH reports receiving speaker fees and/or consultancies from AbbVie, Amgen, AstraZeneca, Boehringer Ingelheim, Bristol Myers Squibb, Chugai, GSK, InflaRx, Janssen, MSD, Novartis, Pfizer, Phadia, Roche, and CSL Vifor. CP reports receiving consulting and speaker fees from GSK, Otsuka, Pfizer, and Roche; grants and speaker or advisory board fees from Roche; has served on advisory boards for AstraZeneca, GSK, and Otsuka; and has received educational grants from GSK, Otsuka, and Pfizer. US reports receiving consulting fees from Amgen, Argenx, AstraZeneca, Boehringer Ingelheim, and CSL Vifor; and research grants from Amgen, AstraZeneca, Bristol Myers Squibb, Genentech, GSK, NorthStar Medical Radioisotopes, NS Pharma, and Novartis. MEW reports receiving consulting, advisory, or speaking honoraria from Allakos, Amgen, Areteia Therapeutics, Arrowhead Pharmaceutical, AstraZeneca, Avalo Therapeutics, Celldex, Connect Biopharma, Eli Lilly, Equillium, GSK, Incyte, Kinaset, Kymera, Merck, Phylaxis, Pulmatrix, Rapt Therapeutics, Recludix Pharma, Regeneron Pharmaceuticals, Roche/Genentech, Sanofi/Genzyme, Sentien, Sound Biologics, Tetherex Pharmaceuticals, Uniquity Bio, Upstream Bio, Verona Pharma, and Zurabio. DB and AH report no disclosures. CR, JH, JNH, EP, and JB are employees of IQVIA, which received funding from AstraZeneca for this research. VHS, LBS, SN, and CNH are or were employees of AstraZeneca at the time the work was conducted and may own stock/stock options.

## Appendix Supplementary materials

Image, application 1

## Footnotes

Supplementary material associated with this article can be found in the online version at doi:10.1016/j.ero.2025.08.009.
