# Supplementary material for: Patients’ perspectives of eosinophilic granulomatosis with polyangiitis within the context of a clinical trial
Source: EULAR Rheumatol Open. 2025 Oct 24;1(4):339–48. doi: 10.1016/j.ero.2025.08.009 (PMC13292453; doi:10.1016/j.ero.2025.08.009)
Supplement: Supplementary file 1 [file mmc1.docx]

**Supplementary Material**

**Patients’ perspectives of eosinophilic granulomatosis with polyangiitis within the context of a clinical trial**

**Table of contents**

[Supplementary Methods 2](#_Ref14105080)

[**Coding and analysis** 2](#_Ref14252018)

[**Data management and transcription** 4](#_Ref14605951)

[**Institutional Review Board approval** 5](#_Ref14795195)

[**Topics discussed in the qualitative interview sub-study** 6](#_Ref14499632)

[**COnsolidated criteria for REporting Qualitative research (COREQ)** 9](#_Ref14975234)

[**Patient research partner contributions** 11](#_Ref14795899)

[Supplementary Table 1. Full demographics and baseline characteristics 12](#_Ref14863091)

[Supplementary Table 2. 6-item Asthma Control Questionnaire scores 15](#_Ref14594935)

[Supplementary Table 3. SF-36v2 scores 16](#_Ref14115376)

[Supplementary Table 4. Daily dose of oral GCs (prednisolone/prednisone equivalent) in mg 22](#_Ref14546912)

[Supplementary Table 5. Patient-reported symptoms and bothersomeness ratings at Interview 1 23](#_Ref14210793)

[Supplementary Table 6. Patient-reported impacts and disturbance ratings at Interview 1 25](#_Ref14760743)

[Supplementary Table 7. Patient-reported symptoms and bothersomeness ratings at Interview 2 27](#_Ref14626681)

[Supplementary Table 8. Patient-reported impacts and disturbance ratings at Interview 2 29](#_Ref14240631)

[Supplementary Table 9. Most frequently mentioned symptoms and impacts, and example quotes 31](#_Ref14548706)

[Supplementary Table 10. Improvements in symptoms and impacts at Interview 2 from Interview 1, and example quotes 43](#_Ref14357335)

[Supplementary Table 11. Primary and secondary outcomes 52](#_Ref14112805)

[Supplementary Figure 1. Design of the qualitative sub-study of MANDARA. R, randomisation. 53](#_Ref14722024)

[Supplementary Figure 2. Number of participants in each interview. 54](#_Ref14235441)

[References 55](#_Ref14206326)

## Supplementary Methods

### **Coding and analysis**

- The goal of coding the data was to facilitate the identification of concepts (e.g. symptoms and impact) that were most important and relevant to participants.
- The de-identified transcripts generated from audio recordings were coded, and the data were organised using qualitative analysis software (MAXQDA 2022™, VERBI Software GmbH, Berlin, Germany) for analysis and reporting.
- There were two coding frameworks (one for the Interview 1 transcripts and one for the Interview 2 transcripts) based on topics in the discussion guides (deductive coding), while the emergence of new concepts from the interviews was also documented by adding codes to the coding frameworks as coding proceeded (inductive coding).
- When new concepts emerged, they were tracked by each coder in-process and discussed at weekly coding meetings. When a new concept was independently mentioned by three different participants, it was added as a new code; if it was mentioned by fewer than three participants, it was coded using the ‘Other symptom’ or ‘Other impact’ code.
- In addition to weekly meetings, alignment meetings ensured that coding frameworks remained up to date, and all updated codes were discussed and appropriately considered. The coding process and discrepancies were discussed during meetings with the coding lead(s) to reconcile and finalise coding for analysis.
- Six members of the IQVIA data analysis team were involved in the coding of transcripts; double coding was completed to ensure consistency and quality of coding among coders for 20% of the transcripts for both interviews.

### **Data management and transcription**

Data management was performed in accordance with the Standard Operating Procedures (SOPs) of IQVIA, a brief description of these processes is provided below.

- After interview completion, IQVIA downloaded and saved audio-recordings from the conferencing vendor platform (i.e., Webex and Mercuri) into an IQVIA secure server in an access-controlled file (ELVIS). Audio-recordings were shared with the transcription vendor (River Mist) via a secure file transfer platform (MoveIT).
- The following quality control (QC) processes were performed: the audio files were transcribed in the language in which the interview was conducted (English, German or French), then the transcription vendor performed the first QC of the transcriptions. The QC review includes redaction of all name identifiers, confirmation of patient study identification number, date, and time. After receiving the QCed interview transcript from the transcription vendor, IQVIA performed a second QC on the transcript, redacting any remaining personal identifiable information (PII) and verifying the quality of the transcript by comparison with the audio recording. For the non-English transcripts (e.g., German and French), the additional steps were followed: transcript in original language was shared with either the translation vendor (TransPerfect) or IQVIA’s internal translation team (IQVIA Translation Services) for translation to English. After receiving the transcript in English, IQVIA verified translation quality of the transcript and performed a third QC which involved comparing the English transcript to the native language transcript.
- The interviews for the sub-study were completed over an approximately 3.5-year period (from December 2019 to June 2023). Over the course of this period, the internal SOPs guiding QC processes were updated and prior to data analysis an updated QC check was performed from July to September of 2023 to ensure the QC process complied with the most current internal SOPs. A tracked version and a clean final version were stored in ELVIS for each interview transcript.
- De-identified responses to discussion questions were captured, via interviewer notes, the audio recording, and/or the written transcript.
- IQVIA stored audio-recordings in a de-identified and encrypted data file until the qualitative interview sub-study “Patients with Eosinophilic Granulomatosis with Polyangiitis (EGPA) Enrolled in Clinical trial MANDARA (D3253C00001)” and analyses were completed, at which time all audio recording were deleted. Data management was conducted in accordance with IQVIA SOPs. All study specific process and definitions are documented by IQVIA.

### **Institutional Review Board approval**

| **Name and address of Institutional Review Board** | **Date of approval** |
| --- | --- |
| EC Hôpital Erasme  Route De Lennik 808  1070 Bruxelles  Belgium | 27 January 2020 |
| Hamilton Integrated Research Ethics Board  (HIREB)  293 Wellington St North, Suite 120  Hamilton, ON L8L 8E7  Canada | 3 June 2020 |
| Advarra  372 Hollandview Trail, Suite 300  Aurora, ON L4G 0A5  Canada | 13 and 18 September 2019 |
| Ethik-Kommission der  Landesärztekammer Baden-  Württemberg  Liebknechtstraße 33  70565 Stuttgart  Germany | 15 January 2020 |
| Ethik-Kommission der Ärztekammer Hamburg  Weidestraße 122b  22083 Hamburg  Germany | 15 January 2020 |
| Ethik-Kommission an der Albert-Ludwigs-Universität  Engelberger Straße 21  79106 Freiburg  Germany | 15 January 2020 |
| Ethik-Kommission der Bayerischen  Landesärztekammer  Mühlbaurstraße 16  81677 München  Germany | 15 January 2020 |
| South West- Central Bristol Research Ethics Committee  Whitefriars, Level 3, Block B, Lewin's Mead  BS1 2NT Bristol  United Kingdom | 20 August 2019 |
| University of Michigan Medical School IRB  2800 Plymouth Road, Building 520  Ann Arbor, MI 48109  United States | 21 January 2020 |
| Mayo Clinic Institutional Review Board  200 First Street Southwest  Rochester, MN 55905  United States | 9 July 2020 |

### **Topics discussed in the qualitative interview sub-study**

| **Topics discussed in Interview 1** | |
| --- | --- |
| **Primary topic** | **Sub-topics** |
| **Demographics and health information** | Age |
|  | Sex/gender |
|  | Race/ethnicity |
|  | Employment |
|  | Education status |
| **EGPA background** | Onset of EGPA symptoms |
|  | Symptoms experienced |
|  | Diagnosis of EGPA |
|  | Symptoms evolved over time since diagnosis |
|  | Treatment or medicines received for EGPA |
| **Symptoms experienced before the first dose of study treatment** | Detailed descriptions of symptoms experienced (e.g., frequency, severity, duration, location in the body, alleviators, triggers, change in the symptom over time, description of the symptom before the first dose of treatment, etc.) |
|  | Description of a typical day prior to joining the study |
|  | Description of a bad EGPA day prior to joining the study |
|  | Most bothersome symptoms |
|  | Bothersome rating (0–10 scale) of symptoms prior to joining the study |
| **Impacts experienced before the first dose of study treatment** | Detailed description of impacts experienced (e.g. frequency, severity, duration, triggers, description of the impact over time, description of the impact before the first dose of the study treatment, etc.) |
|  | Most bothersome impacts |
|  | Disturbance rating (0–10 scale) of impacts prior to joining the study |
| **Motivations to participate in the trial, expectations of the study treatment, and definition of success** | Experience on study treatment since first dose |
|  | Difference in EGPA symptoms since the first dose of the study treatment |
|  | Variance of symptoms from day to day since the first dose of the study treatment |
|  | Change to symptoms and impacts since first dose (rating scale from –10 to +10, where –10 is as worse as it can possibly get and positive +10 is an extreme improvement, and 0 is no change) |
|  | Difference in EGPA impacts since the first dose of the study treatment |
|  | Motivations to participate in the trial, expectations of the study treatment and the patient’s definition of success |
|  | Reactions/side effects to the study treatment (e.g. unexpected effects) |
|  | Impact of COVID-19 on study treatment and EGPA symptoms and impacts |

| **Topics discussed in Interview 2** | |
| --- | --- |
| **Primary topic** | **Sub-topics** |
| **Experience with EGPA today** | Symptoms experienced during study treatment (e.g. description of each symptom, frequency over the last week, duration over the last week, severity, location in the body, alleviators, triggers, etc.) |
|  | Bothersome rating (0–10 scale) of symptoms currently |
| **Experience with EGPA since first interview** | Benefits expected from the study treatment |
|  | Difference in EGPA symptoms since the first interview (e.g. difference in EGPA symptoms since the first interview, change in the severity or frequency or timing of the EGPA symptoms, new symptoms experienced since the first interview, etc.) |
|  | Description of a typical day since the first interview |
|  | Variance in the symptoms from day to day since the first interview |
|  | Description of bad EGPA day since the first interview |
|  | Symptoms rating scale (–10 to +10) change since the first interview |
|  | Difference in EGPA impacts since the first interview (e.g. difference in EGPA impacts since the first interview, change in the severity or frequency or timing of the EGPA impacts, new impacts experienced since the first interview, etc.) |
|  | Impacts rating scale (–10 to +10) change since the first interview |
|  | Reactions/side effects to the study treatment (e.g. unexpected effects) |
|  | Comparison of the study treatment with previous treatments |
| **Patient experience on study treatment** | Difference in the amount of treatment benefit experienced within the first 3 weeks after the first dose versus later in the study |
|  | Meaningful aspects of study treatment (e.g. most meaningful effects experienced with study treatment, factors to be considered meaningful effects, etc.) |
|  | Perceptions of success of study treatment, differences in their experiences of the study drug compared to their experiences with other previous treatment(s), and dislikes about the study treatment |
|  | Impact of COVID-19 on study treatment and EGPA symptoms and impacts |

***Topics discussed in the qualitative interview sub-study***

***Topics discussed in the qualitative interview sub-study***

| **Topics discussed in Interview 2 for those participants who did not complete Interview 1** | |
| --- | --- |
| **Primary topic** | **Sub-topics** |
| **Demographics and health information** | Age |
|  | Sex/gender |
|  | Race/ethnicity |
|  | Employment |
|  | Education status |
| **Experience with EGPA today** | Symptoms experienced during study treatment (e.g. description of each symptom, frequency over the last week, duration over the last week, severity, location in the body, alleviators, triggers, etc.) |
|  | Bothersome rating (0– 10 scale) of symptoms currently |
| **Experience with EGPA during clinical trial** | Benefits expected from the study treatment |
|  | Difference in EGPA symptoms during the trial (e.g. difference in EGPA symptoms since joining the trial, change in the severity or frequency or timing of the EGPA symptoms, new symptoms experienced since the first dose of study treatment, etc.) |
|  | Description of a typical day since the first dose |
|  | Variance of the symptoms from day to day since the first dose |
|  | Description of a bad EGPA day since the first dose |
|  | Symptoms rating scale (–10 to +10) change since the first dose |
|  | Difference in EGPA impacts since the first interview (e.g. difference in EGPA impacts since the first dose, change in the severity or frequency or timing of the EGPA impacts, new impacts experienced since the first dose, etc.) |
|  | Impacts rating scale (–10 to +10) change since the first dose |
|  | Reactions/side effects to the study treatment (e.g. unexpected effects) |
|  | Comparison of the study treatment with previous treatments |
| **Patient experience on study treatment** | Difference in the amount of treatment benefit experienced within the first 3 weeks after first dose versus later in the study |
|  | Meaningful aspects of study treatment (e.g. most meaningful effects experienced with study treatment, factors to be considered meaningful effects, etc.) |
|  | Expectations of study treatment and definition of success |
|  | Participant overall experience on study treatment |
|  | Impact of COVID-19 on study treatment and EGPA symptoms and impacts |

Questions could be read verbatim, but moderators could also rephrase questions to ensure patient understanding. As needed, probing questions based on previous research in EGPA were also asked to gather additional detail on specific topics.

COVID-19, coronavirus disease 2019; EGPA, eosinophilic granulomatosis with polyangiitis.

### **COnsolidated criteria for REporting Qualitative research (COREQ)**

| **COREQ checklist** | | **Response** |
| --- | --- | --- |
| ***Personal characteristics*** | | |
| 1. Interviewer/ facilitator | Which author/s conducted the interview or focus group? | There were five moderators involved in this study:   - Caroline Roberts (author) - Hannah Moessinger - Solene Bayet - Kimberly Kelly - France Ginchereau Sowell |
| 2. Credentials | What were the researcher’s credentials? | - Caroline Roberts, MPH, MA - Hannah Moessinger, MS, PhD - Solene Bayet, MSc - Kimberly Kelly, MPA, PhD - France Ginchereau Sowell, MA, PhD |
| 3. Occupation | What was their occupation at the time of the study | Qualitative researchers |
| 4. Gender | Was the researcher male or female? | Female |
| 5. Experience and training | What experience or training did the researcher(s) have? | Each moderator has completed an internal moderator training program and an internal qualitative moderator assessment and qualification program. Finally, each moderator completes project-specific training. |
| ***Relationship with participants*** | | |
| 6. Relationship established | Was a relationship established prior to study commencement? | There was no relationship established with the participant prior to the start of the study. |
| 7. Participant knowledge of the interviewer | What did the participants know about the researcher? E.g., personal goals, reasons for doing the research | No information about the interviewer was shared with the participants beyond the introduction on the day of the interview where the interviewer shared their name. |
| 8. Interviewer characteristics | What characteristics were reported about the interviewer/facilitator? E.g., Bias, assumptions, reasons and interests in the research topic | The participants were informed that a researcher from IQVIA was conducting the interviews, and that the interviewer was not a physician. |
| ***Data analysis*** | | |
| 24. Number of data coders | How many data coders coded the data? | Four coders total and two coding experts providing oversight. Two coders were paired together as a team to code transcripts from interview 1 and were overseen by an expert-level coder who met regularly with the coding team to answer questions, resolve discrepancies, and review coding. A similar process was used for interview 2, with two new individuals serving as the coding team and a new expert-level coder. |
| 25. Description of the coding tree | Did authors provide a description of the coding tree? | Coders and expert coders providing oversight were trained on the coding framework as well as the approach to be taken for adding new codes derived inductively from the data.    The initial coding framework for Interview 1 included codes for symptoms and impacts experienced prior to the clinical trial and during the clinical trial (from first dose to the time of Interview 1 [i.e., after Visit 2]), changes experienced in symptoms and impacts during the clinical trial, motivations to participate in the clinical trial, expectation of the study treatment, and other study treatment-related topics. Codes were also added inductively as they emerged from the interviews; added codes were discussed and agreed among the coding team including the coders and expert coder.    The initial coding framework for Interview 2 included codes for symptoms and impacts experienced during the clinical trial (from Interview 1 to the time of Interview 2 [i.e., after Visit 16]), changes experienced in symptoms and impacts during the trial, reactions/side effects to the study treatment, and the general experience of the participant during the clinical trial. Codes were also added inductively as they emerged from the interviews; added codes were discussed and agreed among the coding team including the coders and expert coder.    The initial coding frameworks were incorporated into the qualitative analysis software, MAXQDA 2022[™](https://urldefense.com/v3/__https:/en.wikipedia.org/wiki/Trademark_symbol__;!!NLFGqXoFfo8MMQ!tynW3b-Kw3ylvjixc_r1AtMNx08--OUO202mbqHl2L9Ve20DTm5rUiI_mUJVnGbeCp45nZU0Ka8iyqMtcc0RdceaN10$), which was then used to code and organize the data from the transcripts for the analysis and reporting. |
| 26. Derivation of themes | Were themes identified in advance or derived from the data? | Two coding frameworks (one for the interview 1 transcripts and one for the interview 2 transcripts) were developed in advance, based on topics in the discussion guides (deductive coding) while also documenting for the emergence of new concepts from the interviews by adding codes to the coding framework, as coding proceeded (inductive coding). |
| 27. Software | What software, if applicable, was used to manage the data? | MAXQDA 2022[™](https://urldefense.com/v3/__https:/en.wikipedia.org/wiki/Trademark_symbol__;!!NLFGqXoFfo8MMQ!tynW3b-Kw3ylvjixc_r1AtMNx08--OUO202mbqHl2L9Ve20DTm5rUiI_mUJVnGbeCp45nZU0Ka8iyqMtcc0RdceaN10$) |
| 28. Participant checking | Did participants provide feedback on the findings? | No. Results from this study were not shared with participants. |

### **Patient research partner contributions**

Patient research partners David Badenoch and Alisa Hartsell are patients with EGPA who were recruited with assistance from the Vasculitis Patient-Powered Research Network. They joined in discussions of interpretation and presentation of results, and drafting of the manuscript and thus qualified for authorship as per the International Committee of Medical Journal Editors (ICMJE) criteria.

## Supplementary Table 1. Full demographics and baseline characteristics

| **Characteristics** |  | **Sub-study**  **Total participants, N=38** | | **Main trial**  **Total participants, N=140** |
| --- | --- | --- | --- | --- |
| **Age, years, mean (SD)** |  | 52 (12) | | 52 (14) |
| **Sex, n (%)** | Female | 27 (71) | | 84 (60) |
| **Race, n (%)** | White | 34 (89) | | 110 (83) |
|  | Other | 3 (8) | | 5 (4) |
|  | Asian | 1 (3) | | 17 (13) |
|  | Black or African American | 0 (0) | | 0 (0) |
| **Country, n (%)** | United Kingdom | 15 (39) | | 18 (13) |
|  | Germany | 11 (29) | | 17 (12) |
|  | Canada | 6 (16) | | 19 (14) |
|  | Belgium | 3 (8) | | 4 (3) |
|  | United States | 3 (8) | | 13 (9) |
|  | France | 0 (0) | | 33 (24) |
|  | Italy | 0 (0) | | 18 (13) |
|  | Israel | 0 (0) | | 10 (7) |
|  | Japan | 0 (0) | | 8 (6) |
| **Level of education, n (%)** | Elementary | 1 (3) | | - |
|  | High school diploma | 2 (5) | | - |
|  | Some college | 4 (11) | | - |
|  | Associate’s degree | 3 (8) | | - |
|  | Bachelor’s degree | 12 (32) | | - |
|  | Some graduate/postgraduate | 1 (3) | | - |
|  | Graduate/postgraduate | 4 (11) | | - |
|  | Other education described^a^ | 4 (11) | | - |
|  | Not reported | 7 (18) | | - |
| **Language, n (%)** | English | 24 (63) | | - |
|  | German | 11 (29) | | - |
|  | French | 3 (8) | | - |
| **Time since diagnosis, months** | Mean (standard deviation) | 79 (85) | | 62 (68) |
|  | Median (minimum, maximum) | 42 (4, 240) | | 34 (1, 456) |
| **Time since diagnosis, n (%)** | <6 months | 3 (8) | | - |
|  | 6–12 months | 9 (24) | | - |
|  | >12 months to 2 years | 3 (8) | | - |
|  | >2 years to 4 years | 3 (8) | | - |
|  | ≤4 years | 18 (47) | | 79 (56) |
|  | >4 years | 16 (42) | | 61 (44) |
|  | Not reported | 4 (11) | | 0 (0) |
| **Treatment group randomisation, n (%)** | Mepolizumab | 24 (63) | | 70 (50) |
|  | Benralizumab | 14 (37) | | 70 (50) |
| **Daily dose of oral glucocorticoids (prednisolone/prednisone equivalent), mg^b^** | Mean (standard deviation) | 10.4 (4.4) | | 11 (5.3) |
| **Treatments and overall experience** | | **Current treatment  n=35^c^** | **Past treatment  n=35^c^** |  |
| **Treatments, n (%)^d^** | Asthma drug | 22 (63) | 17 (49) | - |
|  | Biologic drug | 0 (0) | 1 (3) | - |
|  | Immunosuppressant | 30 (86) | 32 (91) | - |
|  | Steroid (oral and inhaled) | 14 (40) | 24 (69) | - |
|  | Other^e^ | 21 (60) | 25 (71) | - |
| **Overall experience following treatments,  n (%)** | Improved symptoms | 17 (49) | 26 (74) | - |
|  | Worsened symptoms | 2 (6) | 9 (26) | - |
|  | Improved impacts | 4 (11) | 4 (11) | - |
|  | Worsened impacts | 1 (3) | 3 (9) | - |

This sub-study did not aim to balance the sample between treatment groups or any sociodemographic or clinical characteristics.

The treatment history of the full study population can be found in: Wechsler ME, et al. Engl J Med 2024;390(10):911-21.

^a^Teaching certification or qualification, surveyor qualification or medical training (non-degree programme).

^b^Daily dose of oral glucocorticoids was obtained from the clinical trial data for the participants of this sub-study.

^c^Three of the total 38 participants did not provide a response or only completed Interview 2.

^d^Since these data were self-reported by the participants, they may not match the clinical trial data.

^e^Other treatments or therapies included acupuncture, anticoagulants, antibiotics, antimetabolites, blood pressure medication (e.g. amlodipine), calcium, eye drops, hyposensitisation medication for allergies, nasal spray, pain killers, proton-pump inhibitors (e.g. lansoprazole, omeprazole), levothyroxine and vitamin D.

## Supplementary Table 2. 6-item Asthma Control Questionnaire scores

| **Period** | **Summary statistics** | **Total participants (N=38)** | |
| --- | --- | --- | --- |
|  |  | **ACQ-6 score^a^** | **Change from baseline** |
| **Baseline^b^** | n | 38 | - |
|  | Mean | 1.44 | - |
|  | Standard deviation | 1.152 | - |
|  | Median | 1.33 | - |
|  | Minimum | 0.0 | - |
|  | Maximum | 4.5 | - |
| **Week 1–4** | N | 38 | 38 |
|  | Mean | 1.07 | –0.38 |
|  | Standard deviation | 0.802 | 0.842 |
|  | Median | 0.92 | –0.33 |
|  | Minimum | 0.0 | –3.2 |
|  | Maximum | 3.0 | 1.7 |
| **Week 45–48** | N | 38 | 38 |
|  | Mean | 0.79 | –0.65 |
|  | Standard deviation | 0.962 | 0.951 |
|  | Median | 0.50 | –0.38 |
|  | Minimum | 0.0 | –3.3 |
|  | Maximum | 5.1 | 1.0 |
| **Week 49–52** | n | 38 | 38 |
|  | Mean | 0.75 | –0.69 |
|  | Standard deviation | 0.750 | 0.932 |
|  | Median | 0.48 | –0.50 |
|  | Minimum | 0.0 | –3.1 |
|  | Maximum | 3.0 | 1.6 |

^a^6-item Asthma Control Questionnaire scores were obtained from the clinical trial data for the participants of this sub-study.

^b^‘Baseline’ was defined as the last non-missing measurement on or prior to the date of randomisation.

ACQ-6, 6-item Asthma Control Questionnaire.

## Supplementary Table 3. SF-36v2 scores

| **Time point** | **Summary statistics** | **Total participants (N=38)** | |
| --- | --- | --- | --- |
|  |  | **SF-36v2 score^a^** | **Change from baseline^b^** |
| **SF-36v2 subscale and component: Physical Component Summary (PCS)** | | | |
| **Baseline^c^** | n | 38 | - |
|  | Mean | 43.0 | - |
|  | Standard deviation | 10.10 | - |
|  | Median | 42.8 | - |
|  | Minimum | 19 | - |
|  | Maximum | 61 | - |
| **Week 1** | n | 37 | 37 |
|  | Mean | 44.5 | 1.6 |
|  | Standard deviation | 9.90 | 5.35 |
|  | Median | 44.3 | 0.5 |
|  | Minimum | 20 | –5 |
|  | Maximum | 63 | 20 |
| **Week 40** | n | 37 | 37 |
|  | Mean | 45.9 | 2.7 |
|  | Standard deviation | 10.60 | 6.08 |
|  | Median | 48.2 | 1.2 |
|  | Minimum | 15 | –5 |
|  | Maximum | 62 | 23 |
| **Week 52** | n | 35 | 35 |
|  | Mean | 45.4 | 3.1 |
|  | Standard deviation | 10.48 | 7.43 |
|  | Median | 46.3 | 2.2 |
|  | Minimum | 14 | –19 |
|  | Maximum | 63 | 24 |
| **SF-36v2 subscale and component: Mental Component Summary (MCS)** | | | |
| **Baseline^c^** | n | 38 | - |
|  | Mean | 44.9 | - |
|  | Standard deviation | 11.60 | - |
|  | Median | 48.1 | - |
|  | Minimum | 22 | - |
|  | Maximum | 66 | - |
| **Week 1** | n | 37 | 37 |
|  | Mean | 46.3 | 1.5 |
|  | Standard deviation | 9.28 | 8.39 |
|  | Median | 45.1 | 1.0 |
|  | Minimum | 29 | –18 |
|  | Maximum | 64 | 26 |
| **Week 40** | n | 37 | 37 |
|  | Mean | 47.6 | 2.8 |
|  | Standard deviation | 11.37 | 9.95 |
|  | Median | 51.7 | 4.1 |
|  | Minimum | 16 | –21 |
|  | Maximum | 62 | 26 |
| **Week 52** | n | 35 | 35 |
|  | Mean | 49.8 | 5.3 |
|  | Standard deviation | 9.91 | 11.21 |
|  | Median | 52.6 | 4.0 |
|  | Minimum | 20 | –20 |
|  | Maximum | 69 | 33 |
| **SF-36v2 subscale and component: Physical Functioning (PF)** | | | |
| **Baseline^c^** | n | 38 | - |
|  | Mean | 43.9 | - |
|  | Standard deviation | 10.56 | - |
|  | Median | 45.1 | - |
|  | Minimum | 23 | - |
|  | Maximum | 58 | - |
| **Week 1** | n | 37 | 37 |
|  | Mean | 45.3 | 1.7 |
|  | Standard deviation | 10.17 | 5.32 |
|  | Median | 46.0 | 0.0 |
|  | Minimum | 25 | –6 |
|  | Maximum | 58 | 23 |
| **Week 40** | n | 37 | 37 |
|  | Mean | 48.1 | 4.0 |
|  | Standard deviation | 9.03 | 6.88 |
|  | Median | 49.9 | 3.9 |
|  | Minimum | 25 | –6 |
|  | Maximum | 58 | 25 |
| **Week 52** | n | 35 | 35 |
|  | Mean | 47.9 | 4.8 |
|  | Standard deviation | 9.75 | 8.53 |
|  | Median | 49.9 | 3.9 |
|  | Minimum | 23 | –8 |
|  | Maximum | 58 | 27 |
| **SF-36v2 subscale and component: Role Limitations due to Physical Health (RP)** | | | |
| **Baseline^c^** | n | 38 | - |
|  | Mean | 42.3 | - |
|  | Standard deviation | 10.76 | - |
|  | Median | 40.6 | - |
|  | Minimum | 22 | - |
|  | Maximum | 57 | - |
| **Week 1** | n | 37 | 37 |
|  | Mean | 44.4 | 2.1 |
|  | Standard deviation | 9.50 | 5.53 |
|  | Median | 43.9 | 0.0 |
|  | Minimum | 29 | –9 |
|  | Maximum | 57 | 18 |
| **Week 40** | n | 37 | 37 |
|  | Mean | 45.9 | 3.6 |
|  | Standard deviation | 10.32 | 7.76 |
|  | Median | 46.1 | 0.0 |
|  | Minimum | 22 | –13 |
|  | Maximum | 57 | 24 |
| **Week 52** | n | 35 | 35 |
|  | Mean | 45.2 | 3.6 |
|  | Standard deviation | 9.91 | 10.12 |
|  | Median | 43.9 | 2.2 |
|  | Minimum | 24 | –24 |
|  | Maximum | 57 | 24 |
|  |  |  |  |
|  |  |  |  |
| **SF-36v2 subscale and component: Bodily Pain (BP)** | | | |
| **Baseline^c^** | n | 38 | - |
|  | Mean | 47.8 | - |
|  | Standard deviation | 9.75 | - |
|  | Median | 49.8 | - |
|  | Minimum | 21 | - |
|  | Maximum | 61 | - |
| **Week 1** | n | 37 | 37 |
|  | Mean | 48.9 | 1.1 |
|  | Standard deviation | 9.63 | 5.77 |
|  | Median | 49.8 | 0.0 |
|  | Minimum | 21 | –9 |
|  | Maximum | 61 | 16 |
| **Week 40** | n | 37 | 37 |
|  | Mean | 49.1 | 1.5 |
|  | Standard deviation | 9.08 | 6.52 |
|  | Median | 45.9 | 0.0 |
|  | Minimum | 25 | –17 |
|  | Maximum | 61 | 15 |
| **Week 52** | n | 35 | 35 |
|  | Mean | 49.9 | 2.7 |
|  | Standard deviation | 8.78 | 8.83 |
|  | Median | 50.6 | 4.3 |
|  | Minimum | 26 | –23 |
|  | Maximum | 61 | 23 |
| **SF-36v2 subscale and component: General Health Perceptions (GH)** | | | |
| **Baseline^c^** | n | 38 | - |
|  | Mean | 38.2 | - |
|  | Standard deviation | 9.44 | - |
|  | Median | 35.0 | - |
|  | Minimum | 26 | - |
|  | Maximum | 62 | - |
| **Week 1** | n | 37 | 37 |
|  | Mean | 39.0 | 1.1 |
|  | Standard deviation | 9.39 | 4.68 |
|  | Median | 38.9 | 0.0 |
|  | Minimum | 26 | –7 |
|  | Maximum | 65 | 15 |
| **Week 40** | n | 37 | 37 |
|  | Mean | 40.2 | 1.7 |
|  | Standard deviation | 8.58 | 7.11 |
|  | Median | 38.9 | 0.9 |
|  | Minimum | 26 | –14 |
|  | Maximum | 62 | 21 |
| **Week 52** | n | 35 | 35 |
|  | Mean | 40.8 | 3.1 |
|  | Standard deviation | 8.48 | 7.45 |
|  | Median | 38.9 | 1.3 |
|  | Minimum | 26 | –15 |
|  | Maximum | 62 | 23 |
|  |  |  |  |
|  |  |  |  |
| **SF-36v2 subscale and component: Vitality (VT)** | | | |
| **Baseline^c^** | n | 38 | - |
|  | Mean | 44.9 | - |
|  | Standard deviation | 11.04 | - |
|  | Median | 44.7 | - |
|  | Minimum | 26 | - |
|  | Maximum | 64 | - |
| **Week 1** | n | 37 | 37 |
|  | Mean | 46.2 | 1.5 |
|  | Standard deviation | 9.72 | 6.98 |
|  | Median | 47.4 | 2.7 |
|  | Minimum | 26 | –14 |
|  | Maximum | 66 | 22 |
| **Week 40** | n | 37 | 37 |
|  | Mean | 47.5 | 2.4 |
|  | Standard deviation | 10.00 | 8.17 |
|  | Median | 47.4 | 2.7 |
|  | Minimum | 31 | –14 |
|  | Maximum | 61 | 24 |
| **Week 52** | n | 35 | 35 |
|  | Mean | 48.5 | 4.9 |
|  | Standard deviation | 10.27 | 10.86 |
|  | Median | 50.1 | 5.4 |
|  | Minimum | 28 | –22 |
|  | Maximum | 66 | 35 |
| **SF-36v2 subscale and component: Social Functioning (SF)** | | | |
| **Baseline^c^** | n | 38 | - |
|  | Mean | 42.8 | - |
|  | Standard deviation | 12.53 | - |
|  | Median | 44.4 | - |
|  | Minimum | 17 | - |
|  | Maximum | 57 | - |
| **Week 1** | n | 37 | 37 |
|  | Mean | 45.4 | 2.7 |
|  | Standard deviation | 10.73 | 8.45 |
|  | Median | 46.9 | 0.0 |
|  | Minimum | 22 | –20 |
|  | Maximum | 57 | 25 |
| **Week 40** | n | 37 | 37 |
|  | Mean | 46.6 | 4.1 |
|  | Standard deviation | 10.09 | 12.25 |
|  | Median | 46.9 | 0.0 |
|  | Minimum | 22 | –30 |
|  | Maximum | 57 | 30 |
| **Week 52** | n | 35 | 35 |
|  | Mean | 49.0 | 5.8 |
|  | Standard deviation | 8.75 | 10.55 |
|  | Median | 51.8 | 4.9 |
|  | Minimum | 27 | –20 |
|  | Maximum | 57 | 25 |
|  |  |  |  |
|  |  |  |  |
|  |  |  |  |
| **SF-36v2 subscale and component: Role Limitations due to Emotional Problems (RE)** | | | |
| **Baseline^c^** | n | 38 | - |
|  | Mean | 43.8 | - |
|  | Standard deviation | 11.83 | - |
|  | Median | 44.2 | - |
|  | Minimum | 10 | - |
|  | Maximum | 56 | - |
| **Week 1** | n | 37 | 37 |
|  | Mean | 45.3 | 1.7 |
|  | Standard deviation | 9.34 | 10.04 |
|  | Median | 44.2 | 0.0 |
|  | Minimum | 29 | –23 |
|  | Maximum | 56 | 38 |
| **Week 40** | n | 37 | 37 |
|  | Mean | 47.6 | 3.8 |
|  | Standard deviation | 11.01 | 10.83 |
|  | Median | 55.6 | 3.8 |
|  | Minimum | 17 | –27 |
|  | Maximum | 56 | 27 |
| **Week 52** | n | 35 | 35 |
|  | Mean | 48.9 | 5.6 |
|  | Standard deviation | 9.35 | 10.85 |
|  | Median | 55.6 | 3.8 |
|  | Minimum | 21 | –11 |
|  | Maximum | 56 | 27 |
| **SF-36v2 subscale and component: Mental Health (MH)** | | | |
| **Baseline^c^** | n | 38 | - |
|  | Mean | 45.9 | - |
|  | Standard deviation | 9.45 | - |
|  | Median | 47.8 | - |
|  | Minimum | 21 | - |
|  | Maximum | 63 | - |
| **Week 1** | n | 37 | 37 |
|  | Mean | 46.7 | 1.0 |
|  | Standard deviation | 7.65 | 7.23 |
|  | Median | 47.8 | 0.0 |
|  | Minimum | 35 | –22 |
|  | Maximum | 60 | 17 |
| **Week 40** | n | 37 | 37 |
|  | Mean | 47.9 | 2.1 |
|  | Standard deviation | 9.34 | 8.65 |
|  | Median | 50.3 | 2.5 |
|  | Minimum | 21 | –22 |
|  | Maximum | 63 | 17 |
| **Week 52** | n | 35 | 35 |
|  | Mean | 49.4 | 4.1 |
|  | Standard deviation | 8.87 | 10.53 |
|  | Median | 52.8 | 4.9 |
|  | Minimum | 26 | –25 |
|  | Maximum | 60 | 37 |

^a^SF-36v2 scores were obtained from the clinical trial data for the participants of this sub-study.

^b^Responder threshold values were defined as a significant change and a baseline follow-up correlation of 0.10 was assumed, using an 80% confidence interval (User’s Manual for the SF-36v2 Health Survey, Third Edition)^1^: PCS, 3.4; MCS, 4.6; PF, 4.3; RP, 3.4; BP, 6.2; GH, 7.2; VT, 6.2; SF, 6.9; RE, 4.5; MH, 6.2.

^c^‘Baseline’ was defined as the last non-missing measurement on or prior to the date of randomisation.

BP, Bodily Pain; GH, General Health Perceptions; MCS, Mental Component Summary;
MH, Mental Health; PCS, Physical Component Summary; PF, Physical Functioning;
RE, Role Limitations due to Emotional Problems; RP, Role Limitations due to Physical Health;
SF, Social Functioning; SF-36v2, Short Form 36 Health Survey Questionnaire version 2;
VT, Vitality.

## Supplementary Table 4. Daily dose of oral GCs (prednisolone/prednisone equivalent) in mg

| **Time point** | **Summary statistic** | **Average daily dose  of prednisolone/  prednisone, mg^a^** | **Change from baseline, mg** | **Percentage change from baseline** |
| --- | --- | --- | --- | --- |
| **Baseline^b^** | n | 38 | - | - |
|  | Mean | 10.39 | - | - |
|  | Standard deviation | 4.415 | - | - |
|  | Median | 10.00 | - | - |
|  | Minimum | 7.5 | - | - |
|  | Maximum | 25.0 | - | - |
| **Week 1–4** | n | 38 | 38 | 38 |
|  | Mean | 10.50 | 0.11 | 1.09 |
|  | Standard deviation | 4.524 | 1.014 | 10.143 |
|  | Median | 9.50 | 0.00 | 0.00 |
|  | Minimum | 7.4 | –2.2 | –22.3 |
|  | Maximum | 25.0 | 5.7 | 57.1 |
| **Week 45–48** | n | 38 | 38 | 38 |
|  | Mean | 2.68 | –7.71 | –71.77 |
|  | Standard deviation | 2.750 | 5.130 | 32.362 |
|  | Median | 2.29 | –7.50 | –80.00 |
|  | Minimum | 0.0 | –22.4 | –100.0 |
|  | Maximum | 10.0 | 1.6 | 21.4 |
| **Week 49–52** | n | 38 | 38 | 38 |
|  | Mean | 2.46 | –7.93 | –74.52 |
|  | Standard deviation | 2.645 | 4.844 | 29.988 |
|  | Median | 1.74 | –7.50 | –87.53 |
|  | Minimum | 0.0 | –22.7 | –100.0 |
|  | Maximum | 10.0 | 1.5 | 20.0 |

^a^Daily doses of oral GCs were obtained from the clinical trial data for the 38 participants of this
sub-study.

^b^Baseline is defined as the last non-missing measurement on or prior to the date of randomisation.

GCs, glucocorticoids; SD, standard deviation.

## Supplementary Table 5. Patient-reported symptoms and bothersomeness ratings at Interview 1

| **Symptoms** | **Total mentions (n=35)** | | **Bothersomeness rating^a^** | | | |
| --- | --- | --- | --- | --- | --- | --- |
|  | **n** | **%** | **Participants providing a rating, n** | **ADR** | **Minimum** | **Maximum** |
| **Energy-related symptoms** | | |  |  |  |  |
| Fatigue | 28 | 80.0 | 27 | 7.2 | 2.0 | 10.0 |
| Weakness | 23 | 65.7 | 20 | 7.2 | 3.0 | 10.0 |
| **General** | | |  |  |  |  |
| Other symptoms^b^ | 18 | 51.4 | 7 | 7.2 | 3.0 | 10.0 |
| Difficulty with sense of taste or smell | 14 | 40.0 | 7 | 7.4 | 3.0 | 10.0 |
| Fever | 8 | 22.9 | 8 | 7.0 | 2.0 | 10.0 |
| Dizziness | 6 | 17.1 | 4 | 5.0 | 2.0 | 10.0 |
| Reduction in bone density^c^ | 6 | 17.1 | 1 | 4.0 | 4.0 | 4.0 |
| Dryness of the mouth, throat or nose | 4 | 11.4 | 1 | 4.0 | 4.0 | 4.0 |
| **Hearing-related symptoms** | | |  |  |  |  |
| Hearing loss | 3 | 8.6 | 1 | 10.0 | 10.0 | 10.0 |
| Tinnitus | 2 | 5.7 | 1 | 8.0 | 8.0 | 8.0 |
| **Heart-related symptoms** | | |  |  |  |  |
| Deterioration of heart condition^c^ | 4 | 11.4 | 1 | 10.0 | 10.0 | 10.0 |
| Cardiac rhythm disturbances | 3 | 8.6 | 3 | 8.3 | 6.0 | 10.0 |
| **Nasal symptoms** | | |  | | | |
| Nasal congestion or discharge | 29 | 82.9 | 28 | 6.6 | 2.0 | 10.0 |
| Sinusitis or sinus pressure | 14 | 40.0 | 7 | 6.6 | 3.0 | 10.0 |
| Nasal polyps^c^ | 5 | 14.3 | 2 | 8.5 | 7.0 | 10.0 |
| Nasally voice or voice changes | 3 | 8.6 | 1 | 10.0 | 10.0 | 10.0 |
| Nosebleeds | 3 | 8.6 | 1 | 7.0 | 7.0 | 7.0 |
| Postnasal drip | 3 | 8.6 | 2 | 4.5 | 2.0 | 7.0 |
| Sneezing | 2 | 5.7 | 2 | 4.5 | 3.0 | 6.0 |
| **Neuropathic symptoms** |  |  |  |  |  |  |
| Neuropathy or numbness or tingling | 26 | 74.3 | 21 | 5.3 | 1.0 | 10.0 |
| **Pain** |  |  |  |  |  |  |
| Joint pain | 16 | 45.7 | 13 | 5.2 | 1.0 | 9.0 |
| Muscle pain | 15 | 42.9 | 13 | 5.4 | 2.0 | 10.0 |
| Stomach pain | 11 | 31.4 | 8 | 6.4 | 2.0 | 10.0 |
| Other types of pain^d^ | 9 | 25.7 | 5 | 7.8 | 5.0 | 10.0 |
| Sinus pain | 9 | 25.7 | 8 | 6.1 | 1.0 | 10.0 |
| Cramping | 7 | 20.0 | 5 | 7.0 | 5.0 | 9.0 |
| Headache | 7 | 20.0 | 4 | 8.0 | 5.0 | 9.0 |
| **Respiratory** |  |  |  |  |  |  |
| Difficulty breathing or shortness of breath | 31 | 88.6 | 26 | 6.6 | 2.0 | 10.0 |
| Coughing | 26 | 74.3 | 23 | 6.2 | 2.0 | 10.0 |
| Wheezing | 24 | 68.6 | 19 | 5.6 | 1.0 | 10.0 |
| Chest pain or tightness | 11 | 31.4 | 5 | 6.6 | 3.0 | 10.0 |
| Chest infection or pneumonia^c^ | 8 | 22.9 | 0 | 0.0 | 0.0 | 0.0 |
| **Skin-related symptoms** |  |  |  |  |  |  |
| Skin manifestations | 18 | 51.4 | 18 | 5.6 | 0.0 | 10.0 |
| Bruising^c^ | 7 | 20.0 | 2 | 7.5 | 7.0 | 8.0 |
| Thinning of the skin | 5 | 14.3 | 2 | 9.0 | 9.0 | 9.0 |
| Thrush in mouth or oral thrush | 4 | 11.4 | 0 | 0.0 | 0.0 | 0.0 |
| **Stomach-related symptoms** |  |  |  |  |  |  |
| Irregular bowel movement (alternating constipation and diarrhoea) | 6 | 17.1 | 3 | 8.0 | 7.0 | 10.0 |
| Nausea and vomiting | 3 | 8.6 | 2 | 3.0 | 3.0 | 3.0 |
| **Swelling-related symptoms** |  |  |  |  |  |  |
| Swelling | 6 | 17.1 | 0 | 0.0 | 0.0 | 0.0 |
| Fluid retention^c^ | 3 | 8.6 | 1 | 10.0 | 10.0 | 10.0 |
| **Weight-related symptoms** |  |  |  |  |  |  |
| Weight gain | 12 | 34.3 | 3 | 7.7 | 3.0 | 10.0 |
| Weight loss | 7 | 20.0 | 4 | 4.0 | 0.0 | 6.0 |

^a^Bothersomeness ratings were assessed on an ADR scale (0–10, where 0 is not bothersome at all and 10 is extremely bothersome). ADRs were based on the number of participants who provided a quantitative rating, which was not always the same as the number of participants who endorsed the symptom. Some participants provided qualitative descriptions and, despite gentle encouragement from the interviewer, did not provide a quantitative number.

^b^Other symptoms (as reported by the participants) included vision disturbance, blood clot, urinary difficulty, coughing up blood, difficulty eating, congested throat, tendonitis, muscle atrophy or muscle loss or wasting, ear infections, appetite loss, sweating, fluid or phlegm from lungs, cyst in the throat and salivary gland swelling.

^c^Responses that participants provided in relation to a question about symptoms.

^d^Other types of pain (as described by the participants) included pain in the soles of the feet, nerve pain, spinal pain, neck pain, back pain, abdominal pain, heart pain, bone pain and generalised pain.

ADR, average disturbance rating.

## Supplementary Table 6. Patient-reported impacts and disturbance ratings at Interview 1

| **Impacts** | **Total mentions (n=35)** | | **Disturbance rating^a^** | | | |
| --- | --- | --- | --- | --- | --- | --- |
|  | **n** | **%** | **Participants providing a rating, n** | **ADR** | **Minimum** | **Maximum** |
| **Cognitive** | | |  | | | |
| Cognitive impairment (memory loss, difficulty concentrating) | 9 | 25.7 | 8 | 8.9 | 4.0 | 10.0 |
| **Daily activities** | | |  |  |  |  |
| Difficulty with daily or everyday activities | 17 | 48.6 | 15 | 8.2 | 4.0 | 10.0 |
| Ability to drive | 4 | 11.4 | 0 | 0.0 | 0.0 | 0.0 |
| Ability to travel | 4 | 11.4 | 2 | 6.3 | 5.0 | 8.0 |
| **Emotional or psychological** | | |  |  |  |  |
| Embarrassment or impaired confidence | 9 | 25.7 | 4 | 7.8 | 5.0 | 10.0 |
| Depression | 8 | 22.9 | 4 | 8.5 | 8.0 | 9.0 |
| Mood swings | 7 | 20.0 | 2 | 10.0 | 10.0 | 10.0 |
| Feeling nervous or anxious | 6 | 17.1 | 3 | 5.3 | 1.0 | 9.0 |
| Sadness | 5 | 14.3 | 3 | 6.7 | 3.0 | 9.0 |
| Fear or worry | 4 | 11.4 | 3 | 9.7 | 9.0 | 10.0 |
| Feeling frustration | 4 | 11.4 | 0 | 0.0 | 0.0 | 0.0 |
| Feeling uncomfortable | 3 | 8.6 | 1 | 10.0 | 10.0 | 10.0 |
| Hope for future or positive outlook | 0 | 0.0 | 0 | 0.0 | 0.0 | 0.0 |
| **General** | | |  |  |  |  |
| Quality and quantity of sleep | 25 | 71.4 | 18 | 6.9 | 2.0 | 10.0 |
| Other impacts^b^ | 22 | 62.9 | 13 | 7.9 | 4.0 | 10.0 |
| **Physical** |  |  |  |  |  |  |
| Ability to exercise or engage in more strenuous activities | 26 | 74.3 | 15 | 7.8 | 2.0 | 10.0 |
| Difficulty walking | 19 | 54.3 | 7 | 6.6 | 3.0 | 10.0 |
| Difficulty climbing stairs or going uphill | 15 | 42.9 | 1 | 5.5 | 5.0 | 6.0 |
| Reduced strength | 6 | 17.1 | 0 | 0.0 | 0.0 | 0.0 |
| Ability to lift and carry things | 4 | 11.4 | 0 | 0.0 | 0.0 | 0.0 |
| **Role at work** |  |  |  |  |  |  |
| Ability to work | 22 | 62.9 | 19 | 8.2 | 3.0 | 10.0 |
| **Social or relationships** |  |  |  |  |  |  |
| Ability to engage in social activities | 21 | 60.0 | 16 | 6.0 | 0.0 | 9.0 |
| Parenting or ability to look after children | 5 | 14.3 | 4 | 8.5 | 7.0 | 10.0 |
| Effect of disease on partners or dependents | 2 | 5.7 | 1 | 10.0 | 10.0 | 10.0 |

^a^Disturbance ratings were assessed on an ADR scale (0–10, where 0 is not bothersome at all and 10 is extremely bothersome). ADRs were based on the number of participants who provided a quantitative rating, which was not always the same as the number of participants who endorsed the impact. Some participants provided qualitative descriptions and, despite gentle encouragement from the interviewer, did not provide a quantitative number.

^b^Other impacts (reported by ≤3 participants) were grouped as ‘other impacts’ and included (as reported by the participants) difficulty planning vacations; being careful about what to eat; restlessness; impact on leisure activities or hobbies; feeling stressed, upset, ill-tempered or in a bad mood; difficulty with motor skills; voice loss; increase in medical appointments; difficulty holding a conversation; impaired quality of life; financial impact; missing out on life experiences; falling; and mental exhaustion.

ADR, average disturbance rating.

## Supplementary Table 7. Patient-reported symptoms and bothersomeness ratings at Interview 2

| **Symptoms** | **Total mentions (n=32)** | | **Bothersomeness rating^a^** | | | |
| --- | --- | --- | --- | --- | --- | --- |
|  | **n** | **%** | **Participants providing rating, n** | **ADR** | **Minimum** | **Maximum** |
| **Energy-related symptoms** | | |  |  |  |  |
| Fatigue | 22 | 68.8 | 12 | 6.9 | 2.0 | 10.0 |
| Weakness | 17 | 53.1 | 11 | 4.4 | 1.0 | 9.0 |
| **General** | | |  |  |  |  |
| Difficulty with sense of taste or smell | 12 | 37.5 | 6 | 5.0 | 1.0 | 10.0 |
| Other symptoms^b^ | 12 | 37.5 | 4 | 8.8 | 7.0 | 10.0 |
| Dizziness | 4 | 12.5 | 1 | 9.0 | 9.0 | 9.0 |
| Fever | 6 | 18.8 | 2 | 3.5 | 3.0 | 4.0 |
| Reduction in bone density^c^ | 1 | 3.1 | 0 | 0.0 | 0.0 | 0.0 |
| Dryness of the mouth, throat or nose | 0 | 0 | 0 | 0.0 | 0.0 | 0.0 |
| **Hearing-related symptoms** | | |  |  |  |  |
| Tinnitus | 3 | 9.4 | 2 | 4.0 | 4.0 | 4.0 |
| Hearing loss | 1 | 3.1 | 0 | 0.0 | 0.0 | 0.0 |
| **Heart-related symptoms** | | |  |  |  |  |
| Cardiac rhythm disturbances | 4 | 12.5 | 1 | 3.0 | 3.0 | 3.0 |
| Deterioration of heart condition^c^ | 0 | 0 | 0 | 0.0 | 0.0 | 0.0 |
| **Nasal symptoms** | | |  | | | |
| Nasal congestion or discharge | 27 | 84.4 | 22 | 4.2 | 0.0 | 10.0 |
| Sinusitis or sinus pressure | 10 | 31.3 | 5 | 7.2 | 5.0 | 10.0 |
| Sneezing | 6 | 18.8 | 2 | 7.0 | 7.0 | 7.0 |
| Nosebleeds | 4 | 12.5 | 3 | 4.7 | 3.0 | 6.0 |
| Postnasal drip | 4 | 12.5 | 2 | 6.0 | 5.0 | 7.0 |
| Nasal polyps^c^ | 3 | 9.4 | 0 | 0.0 | 0.0 | 0.0 |
| Nasally voice or voice changes | 1 | 3.1 | 1 | 5.0 | 5.0 | 5.0 |
| **Neuropathic symptoms** |  |  |  |  |  |  |
| Neuropathy or numbness or tingling | 15 | 46.9 | 11 | 3.9 | 1.0 | 10.0 |
| **Pain** |  |  |  |  |  |  |
| Joint pain | 12 | 37.5 | 10 | 4.2 | 2.0 | 8.0 |
| Muscle pain | 10 | 31.3 | 9 | 5.8 | 0.0 | 10.0 |
| Other types of pain^d^ | 9 | 28.1 | 3 | 6.7 | 2.0 | 8.0 |
| Stomach pain | 8 | 25.0 | 5 | 4.6 | 1.0 | 8.0 |
| Headache | 6 | 18.8 | 5 | 5.0 | 3.0 | 8.0 |
| Sinus pain | 4 | 12.5 | 4 | 2.0 | 0.0 | 3.0 |
| Cramping | 2 | 6.3 | 2 | 4.5 | 1.0 | 8.0 |
|  |  |  |  |  |  |  |
|  |  |  |  |  |  |  |
| **Respiratory** |  |  |  |  |  |  |
| Difficulty breathing or shortness of breath | 27 | 84.4 | 16 | 4.9 | 0.0 | 10.0 |
| Coughing | 18 | 56.3 | 13 | 2.8 | 0.0 | 8.0 |
| Wheezing | 10 | 31.3 | 8 | 1.3 | 0.0 | 3.0 |
| Chest pain or tightness | 7 | 21.9 | 0 | 0.0 | 0.0 | 0.0 |
| Chest infection or pneumonia^c^ | 6 | 18.8 | 2 | 4.5 | 4.0 | 5.0 |
| **Skin-related symptoms** |  |  |  |  |  |  |
| Skin manifestations | 14 | 43.8 | 11 | 4.5 | 1.0 | 8.0 |
| Thinning of the skin | 2 | 6.3 | 0 | 0.0 | 0.0 | 0.0 |
| Bruising^c^ | 2 | 6.3 | 1 | 0.0 | 0.0 | 0.0 |
| Thrush in mouth or oral thrush | 0 | 0 | 0 | 0.0 | 0.0 | 0.0 |
| **Stomach-related symptoms** |  |  |  |  |  |  |
| Irregular bowel movement (alternating constipation and diarrhoea) | 5 | 15.6 | 3 | 8.3 | 7.0 | 10.0 |
| Nausea and vomiting | 5 | 15.6 | 1 | 8.0 | 8.0 | 8.0 |
| **Swelling-related symptoms** |  |  |  |  |  |  |
| Fluid retention^c^ | 5 | 15.6 | 1 | 3.0 | 3.0 | 3.0 |
| Swelling | 1 | 3.1 | 1 | 0.0 | 0.0 | 0.0 |
| **Weight-related symptoms** |  |  |  |  |  |  |
| Weight loss | 10 | 31.3 | 5 | 4.8 | 0.0 | 7.0 |
| Weight gain | 7 | 21.9 | 3 | 9.7 | 9.0 | 10.0 |

^a^Bothersomeness ratings were assessed on an ADR scale (0–10, where 0 is not bothersome at all and 10 is extremely bothersome). ADRs were based on the number of participants who provided a quantitative rating, which was not always the same as the number of participants who endorsed the symptom. Some participants provided qualitative descriptions and, despite gentle encouragement from the interviewer, did not provide a quantitative number.

^b^Other symptoms (as reported by the participants) included vision disturbance, blood clot, urinary difficulty, coughing up blood, difficulty eating, congested throat, tendonitis, muscle atrophy or muscle loss or wasting, ear infections, appetite loss, sweating, fluid or phlegm from lungs, cyst in the throat and salivary gland swelling.

^c^Responses that participants provided in relation to a question about symptoms.

^d^Other types of pain (as described by the participants) included pain in the soles of the feet, nerve pain, spinal pain, neck pain, back pain, abdominal pain, heart pain, bone pain and generalised pain.

ADR, average disturbance rating.

## Supplementary Table 8. Patient-reported impacts and disturbance ratings at Interview 2

| **Impacts** | **Total mentions (n=32)** | | **Disturbance rating^a^** | | | |
| --- | --- | --- | --- | --- | --- | --- |
|  | **n** | **%** | **Participants providing a rating, n** | **ADR** | **Minimum** | **Maximum** |
| **Cognitive** | | |  | | | |
| Cognitive impairment (memory loss, difficulty concentrating) | 6 | 18.8 | 3 | 3.0 | 2.0 | 5.0 |
| **Daily activities** | | |  |  |  |  |
| Difficulty with daily or everyday activities | 20 | 62.5 | 11 | 4.3 | 0.0 | 7.0 |
| Ability to drive | 4 | 12.5 | 1 | 2.0 | 2.0 | 2.0 |
| Ability to travel | 2 | 6.3 | 0 | 0.0 | 0.0 | 0.0 |
| **Emotional or psychological** | | |  |  |  |  |
| Fear or worry | 11 | 34.4 | 3 | 2.3 | 0.0 | 7.0 |
| Hope for future or positive outlook | 8 | 25.0 | 0 | 0.0 | 0.0 | 0.0 |
| Depression | 3 | 9.4 | 0 | 0.0 | 0.0 | 0.0 |
| Feeling nervous or anxious | 3 | 9.4 | 0 | 0.0 | 0.0 | 0.0 |
| Mood swings | 3 | 9.4 | 1 | 5.0 | 5.0 | 5.0 |
| Embarrassment or impaired confidence | 2 | 6.3 | 1 | 7.0 | 7.0 | 7.0 |
| Feeling frustration | 1 | 3.1 | 0 | 0.0 | 0.0 | 0.0 |
| Sadness | 1 | 3.1 | 0 | 0.0 | 0.0 | 0.0 |
| Feeling uncomfortable | 0 | 0 | 0 | 0.0 | 0.0 | 0.0 |
| **General** | | |  |  |  |  |
| Quality and quantity of sleep | 26 | 81.3 | 11 | 3.7 | 0.0 | 10.0 |
| Other impacts^b^ | 7 | 21.9 | 4 | 2.9 | 0.0 | 5.0 |
| **Physical** |  |  |  |  |  |  |
| Ability to exercise or engage in more strenuous activities | 25 | 78.1 | 8 | 4.1 | 0.0 | 8.0 |
| Difficulty walking | 17 | 53.1 | 4 | 4.3 | 0.0 | 7.0 |
| Ability to lift and carry things | 7 | 21.9 | 2 | 5.5 | 5.0 | 6.0 |
| Difficulty climbing stairs or going uphill | 5 | 15.6 | 0 | 0.0 | 0.0 | 0.0 |
| Reduced strength | 0 | 0 | 0 | 0.0 | 0.0 | 0.0 |
| **Role at work** |  |  |  |  |  |  |
| Ability to work | 17 | 53.1 | 8 | 5.1 | 2.0 | 10.0 |
| **Social or relationships** |  |  |  |  |  |  |
| Ability to engage in social activities | 20 | 62.5 | 10 | 3.1 | 0.0 | 9.0 |
| Parenting or ability to look after children | 5 | 15.6 | 1 | 10.0 | 10.0 | 10.0 |
| Effect of disease on partners or dependents | 4 | 12.5 | 0 | 0.0 | 0.0 | 0.0 |

^a^Disturbance ratings were assessed on an ADR scale (0–10, where 0 is not bothersome at all and 10 is extremely bothersome). ADRs were based on the number of participants who provided a quantitative rating, which was not always the same as the number of participants who endorsed the impact. Some participants provided qualitative descriptions and, despite gentle encouragement from the interviewer, did not provide a quantitative number.

^b^Other impacts (reported by ≤3 participants) were grouped as ‘other impacts’ and included (as reported by the participants) difficulty planning vacations; being careful about what to eat; restlessness; impact on leisure activities or hobbies; feeling stressed, upset, ill-tempered or in a bad mood; difficulty with motor skills; voice loss; increase in medical appointments; difficulty holding a conversation; impaired quality of life; financial impact; missing out on life experiences; falling; and mental exhaustion.

ADR, average disturbance rating.

## Supplementary Table 9. Most frequently mentioned symptoms and impacts, and example quotes

| **Symptoms** | | **Example quotes^a^** | |
| --- | --- | --- | --- |
| **Interview 1,  number of mentions, % of total participants (n=35)** | **Interview 2,  number of mentions, % of total participants (n=32)** | **Interview 1** | **Interview 2** |
| **Difficulty breathing or shortness of breath**  n=31, 89% | **Difficulty breathing or shortness of breath**  n=27, 84% | *‘The main symptoms of late have been…well, relating to my chest, shortness of breath, wheezing, and I always have a cough. Always. And it has been productive for quite some months’.* | *‘In the few months or maybe 6 months or so running up to the beginning of the study, my asthma was really bad. I could barely walk 100 meters without getting out of breath.* ***I couldn’t lift a bag of groceries, and I was out of breath going up the stairs to bed, and now I feel normal****’.* |
|  |  | *‘The shortness of breath and cardiac rhythm disturbances are mostly in combination, and* ***it mostly begins with the shortness of breath****. And then, often the cardiac rhythm disturbances develops and this skin rash, which mostly begins a little later’.* | *‘The most significant, this, that the asthma problem, this difficulty breathing, that has gone.* ***That has been the most important for me****’.* |
|  |  | *‘****I find it hard to breathe deeply****. Sometimes I have to really take a deep breath. I have to lift my chest cavity up. It's hard to explain without a video, you know what I mean? But I sort of pull my chest up and take a breath in. Also breathing out is quite…it's a bit crackly when I breathe out’.* | *‘Well, the main one is my asthma. I get quite wheezy at times and difficult to breathe out rather than… That’s the main one’.* |
| **Nasal congestion or discharge**  n=29, 83% | **Nasal congestion or discharge**  n=27, 84% | *‘****It was always the nasal*** *congestion was there. It would maybe be a bit worse in the morning and sort of blowing your nose and getting rid of some mucus and stuff’.* | *‘I guess,* ***if I have a saline rinse, nasal rinse that I use and there’s a medical one and that, so that…I use that to try and relieve the congestion****. If it is really bad with coughing in sort of the back of my throat, then I might use a puffer. I haven’t had to use that a lot in the last while. Like it hasn’t been bad enough to have to use it’.* |
|  |  | *‘[…] just a nasal voice and then pain as I said before. I have no pain, but this pressure, so not pain, but it’s such a* ***massive pressure on certain parts of my face, where my sinuses are. It gets worse and worse****’.* | *‘It doesn’t inhibit me. Let’s put it that way. It doesn’t bother me when I’m trying to do something and focus on something’.* |
|  |  | *‘Every other day, I would say, it would be you’d wake up, blow your nose and then a big nosebleed would start.* ***That’s how you’d start your day, really. It would be congested****. You’d blow your nose, get rid of it and then you’d think, “Where did that come from?” but it was there’.* | *‘You wake up in the morning, and you feel very congested; you feel like you’ve got a blocked-up nose…It goes away, yeah.* ***It’s just like you wake up and you’re so congested****’.*  *‘I don’t know what happens in the middle of the night, but you just feel so congested. And you think, oh gosh, I really…you want to clear your nose and that’s the only way you know how to.* ***And then you get the liquid, you know, sometimes you get the phlegm coming down the back of your throat and it’s like, ugh****’.* |
|  |  | *‘I’ve got mild congestion. A little bit of phlegm. But really… I take puffers before I run and then I’m fine’.* | *‘[…] I mean, I don’t know if you can hear it in my voice, but I’m kind of congested all the time’.* |
| **Fatigue**  n=28, 80% | **Fatigue**  n=22, 69% | *‘[…]* ***I was so tired all the time****. Once I get up to go to work and so on, but I kind of felt exhausted the whole time’.* | *‘Yeah. I must admit I am very tired. The consultant I see at the hospital is doing some vitamin B12 tests at the moment to see if I’m absorbing enough vitamin B12. But yeah, I can go to sleep if I sit down. Yeah, very tired’.* |
|  |  | *‘[…] So in the evenings, I feel pretty tired because* ***I don’t feel like I get a really good night’s sleep’****.* | *‘If I’m doing something like gardening and that I’ve noticed lately starting the gardening,* ***I don’t have a lot of energy; I don’t last very long****. Yeah. Tennis seems to be still okay, I don’t…I’m not playing like top level; I’m an intermediate player, so I’m playing it my usual kind of standard. But I kind of need a day in between to recover’.* |
|  |  | *‘[…] Exhaustion was the number one from the beginning. This exhaustion, they can’t explain it. It’s just you get extremely tired out of nowhere.* ***That was the biggest thing that impacted my life…the fatigue*** *I find, so’.* | *‘On a bad day, like I said, sometimes I’ll wake up still wanting to go back to bed, or totally drained. Or I’ll wake up, I think I have energy, and I’ll start making breakfast, get the kids off to school and* ***then it’s just totally, completely drained, and I have to stay on the couch or I feel weak; I feel exhausted****. Everybody actually can tell. […] I can do a little bit of housework and then I’m done again for the rest of the day’.* |
| **Neuropathy, numbness or tingling**  n=26, 74% | **Neuropathy, numbness or tingling**  n=15, 47% | *‘I get a lot of numbness in my leg. I’ve also got numbness in three of my toes on my left foot and two toes on my right foot. That’s all to do with the joints, I believe. It’s mainly that type of thing, really. And I actually can’t…* ***I haven’t got grip in my arms properly anymore because the joints are getting weaker. So I find it difficult to open jars and so forth…*** *My left leg gets weak … towards the end of the day when I’m getting tired or normal tiredness’.* | *‘Oh, yeah, yeah. My little toe and the toes next to it are always numb. And my legs… Yeah, the weakness in my leg is all the time.* ***It gets worse as the day goes on. I’m very stiff when I first get out of bed in the morning. My joints are very stiff****’.* |
|  |  | *‘[…]* ***very very tight rubber socks on,*** *[…]* ***I think that is what it feels like.*** *But otherwise, I sometimes have a tingling sensation in the hands’.* | *‘Yes [the numbness is there], sometimes, so not always. That’s every now and then’.* |
|  |  | *‘Then it also depends on the level of exertion.* ***If, for example, I was walking a lot, then it is worse****. Especially in the feet’.* | *‘The numbness itself is always there, I would say, but it is, let's say, on three days really more intense, a bit more’.* |
| **Coughing**  n=26, 74% | **Coughing**  n=18, 56% | *‘It’s difficult to stop coughing at night’.* | *‘****As soon as I lie down at night, I start coughing, and I'm quite wheezy then****. Or if I'm walking quite fast, I get a bit wheezy. It's more strenuous to breathe in and out, but particularly out it's… I can support that with, I have an inhaler, which I use’.* |
|  |  | *‘I’ll go through periods where I’m not too bad, and then I’ll have mini relapses of sort of blood,* ***coughing up blood,*** *or blood after bowel movements, but not frequently’.* | *‘I’ve had a few months during the course of the treatment where I’ve woken up feeling sort of a little bit chesty and with a chesty cough, and I’ve reported that as I’ve gone to each appointment. But it’s not been a regular thing.* ***And only, I think, during the trial, I’ve had to use my inhaler probably three or four times****’.* |
|  |  | *‘Not runny nose, but just having to blow your nose really frequently in order to clear it and just getting some* ***fluid running down your throat, which then causes a little bit of coughing.*** *Every now and again, probably get a short burst of coughing, but not that frequently’.* | *‘Rarely do I have coughing at night, only rarely’.* |
|  |  | *‘Yes, I do cough.* ***Sometimes it’s productive, and sometimes it’s not. If I awake in the night, I might be a short of breath and a bit of a dry cough****. Then I have to use my inhaler and it all goes away again’.* | *‘You cough; you wheeze; you try not to cough, and* ***it was a vicious circle****’.* |
| **Wheezing**  n=24, 69% | **Wheezing**  n=10^b^, 31% | *‘[…] Even white wine has done it occasionally and made me very wheezy. I would take an antihistamine tablet or a puff of Ventolin, and that would settle it down’.*  *‘[…] I don’t always hear myself, but my sis…****other people say, “Oh, you are wheezy tonight,”*** *whereas I haven’t noticed’.* | *—^b^* |
| **Weakness**  n=23, 66% | **Weakness**  n=17, 53% | *‘To be honest, I’ve been feeling so weak all the time –* ***when you’re feeling so sick, indescribably weak, weak and sick, without being able to explain why****’.* | *‘I still have this loss of strength’.* |
|  |  | *‘And I actually can’t…I haven’t got grip in my arms properly anymore because the joints are getting weaker.* ***So, I find it difficult to open jars and so forth’.*** | *‘****It takes about 15 minutes or so for my joints to loosen up so that I can actually get about and do what I want to do****’.* |
|  |  | *‘The muscle weakness, my legs; that’s the biggest, and* ***when I walk up my stairs at home, by the time I get to the top, some nights I just feel like there’s nothing left to give, like I can barely walk****. I feel weak like it feels… And now, like I said, I’m starting to feel that muscle weakness in my arms, but the legs have been a problem from day one. […]* ***Like I don’t have the strength to keep my arms up****’.* | *‘I had a lot of muscle joint pains, weakness in my legs.* ***I could barely walk****’.* |
|  |  | *‘My leg has hardly any muscle on it. And when I walk, I thought there would be an improvement after the boot was taken off, but* ***my joints are weak. My ankles are weak. It affects my knees. And even if I walk quite a long way, my hips as well.*** *[…] It’s a weakness in the leg because of the tendon, tendonitis. I’ve still got it. The ankle’s still swollen’.* | *‘I am left-handed and my left hand became a little weak, I would say, about six months ago, or something… For a while, it felt a little bit weak for opening jars and things, or gripping something, but that all has more or less gone away. At first, I didn’t…****I wasn’t able to write as tightly as I used to be. I had to be a little bit careful. But now, it’s okay. I can write normally. There was a little bit of weakness in my left arm, but it’s fine now****’.* |
| **Skin manifestations**  n=18, 51% | **Skin manifestations**  n=13, 41% | *‘I started off with rashes and bruising. My asthma got worse, quite significantly worse. And I was getting joint pain and things like that. They initially thought I had Lyme disease, and then that wasn’t it. Then they thought it was something called Wells’ disease, and it wasn’t that. Then I saw a professor at […] Hospital and he diagnosed the Churg–Strauss’.* | *‘I’ve only had one skin flare-up in the last year, which is quite different…They’re much better. They’re much better. I’ve only had a couple of them, where before I used to get them really badly on my hips and on my legs particularly’.* |
|  |  | *‘[…] I started to get skin nodules. I noticed a couple on my elbow at first. I thought I had just knocked my elbow and cut it. Then there was sort of nodules on my fingers and my hands.’.* | *‘The only other thing is a rash appeared on my left knee and right shin, back in July, August, and that’s…it went on my knee after about a month, but it’s remained on my right lower leg still’.* |

| **Impacts** |  | **Example quotes^a^** | |
| --- | --- | --- | --- |
| **Interview 1,  number of mentions, % of total participants (n=35)** | **Interview 2,  number of mentions, % of total participants (n=32)** | **First Interview** | **Second Interview** |
| **Ability to exercise or engage in strenuous activities**  n=26, 74% | **Ability to exercise or engage in strenuous activities**  n=25, 78% | *‘The physical side of things, as well. I can’t achieve what I used to before, exercise or physically, so* ***I have to limit myself during the day****. If I feel okay, I tend to try to rush around and do stuff, do exercise, or do physical stuff around the house, or whatever.* ***Not realizing I’m going to wipe myself out, so it affects my whole day, really****’.* | *‘So,* ***fatigue’s still an issue, but less so than it was****. Chest…sort of out of breath chestiness, I describe it as, that’s been a fairly consistent issue, sort of each month, where* ***I still get out of breath with very limited activity, physical activity****’.* |
|  |  | *‘Taking risks [is] basically out of the question. I live in a cold weather state where a lot of our winter activities are less than super safe.* ***I had to put away doing any of that. Even something as simple as like going sledding with my son, I have to think real hard about whether or not if I hit a tree or if I slip on the ice, if I break a bone, the consequences could literally kill me.*** *Is that worth the risk of going down the sledding hill or going ice skating or any of the other wacky things we do here in the winter’?* | *‘I don’t hesitate to get up and go up and down the stairs.* ***That used to be something that I tried to avoid because going up and down the stairs in our house, it would inevitably make me short of breath or make me wheeze. Now, I can haul groceries up and down the stairs. I don’t really worry about it.*** *I take the dog for a walk 3 or 4 times a day. Again, it’s not something that I really relished doing before because I never knew how far I could get before I wasn’t breathing very well’.* |
|  |  | *‘I think just kind of the tennis really.* ***I’m much more fatigued than I used to be. I used to be able to play a lot longer’.*** | *‘I'm fairly active, like I still play tennis, 2 or 3 times a week and I try and get out to walk. But if I'm doing something like gardening and that* ***I've noticed lately starting the gardening, I don't have a lot of energy. I don't last very long****. Yeah. Tennis seems to be still okay, I don't…I'm not playing like top level; I'm an intermediate player, so I'm playing it my usual kind of standard. But I kind of need a day in between to recover’.* |
| **Quality and quantity of sleep**  n=25, 71% | **Quality and quantity of sleep**  n=26, 81% | *‘Yes, definitely [have difficulty sleeping], so that* ***I woke up a couple of times at night*** *and had to cough up phlegm’.* | *‘Yes, that’s normal, that’s okay.* ***That was difficult before. I think I often woke up with shortness of breath****, or I don’t remember… Everyone has the problem of not being able to fall asleep, but that's not the case with me. I’m just so exhausted’.* |
|  |  | *‘It was more the congestion and not being able to breathe properly. Not a huge coughing fit or anything. It might be a bit of coughing, but more the nasal congestion,* ***not being able to breathe. So, kind of waking myself up snoring, waking myself up, waking my husband up****’.* | *‘I've been a bit embarrassed by the congestion and sort of how it affects. Because* ***I end up breathing very heavily at night apparently, and that's been a bit just embarrass…more embarrassing than anything else.*** *He’s kind of used to it now. He wakes up and says I've really been breathing heavily’.* |
|  |  | *‘****Really difficult to breathe, keeping me awake at night.*** *I tried over-the-counter medications, but nothing managed to clear it. The doctor prescribed some nasal sprays, which again, I gave it a go for a couple of months, but it just seemed to be getting worse, to be honest’.* | *‘****Previously, I couldn’t breathe. I couldn’t sleep at night because I’d have to stand up to breathe.*** *So, [my nostrils] were almost completely blocked’.* |
|  |  | *‘Oh, definitely.* ***I would wake up several times a night with a tight chest, having to use my inhaler. I was so tired all the time...*** *I get up to go to work and so on, but I kind of felt exhausted the whole time. I wasn’t sleeping properly’.* | *‘Well,* ***I don’t sleep well. I go to bed, and I tend to wake up several times in the night for various reasons, sometimes for no reason at all. I just wake up. So yeah, I think it’s [EGPA] affected me in that respect. I get up and I don’t feel rested****, basically’.* |
| **Ability to work**  n=22, 63% | **Ability to work**  n=17, 53% | *‘It was also very difficult for me to be able to concentrate, so* ***for me to be able to work intellectually on certain things, it took a lot of effort for me****’.* | *‘I have already been affected. I was previously the managing director of two companies. I’ve given that up now, because the physical and mental burden, it’s just too much for me now, to be honest. I have the physical stamina to do certain things, or also the ability to concentrate, but this has also significantly reduced at the end of the day.* ***I already have the feeling that I am no longer performing as well with everything as I was before.*** *Not from concentration, not from endurance, but from memory retention, and things like that.* ***So, there are shortfalls there, and I feel like I can’t really cope in a job like management. It’s just too strenuous for me****, I didn’t want to do that anymore and now I’ve-- well, you have to structure your day somehow and so on. So, of course,* ***I’ve given all that up and resigned****, and now I’m freelance, where I can say to myself, I can allocate my time as I want, I can burden myself as much as I want’.* |
| **Ability to engage in social activities**  n=21, 60% | **Ability to engage in social activities**  n=20, 63% | *‘My social life?* ***From, I think, when I had my really first flare-up, my social life has gone to zero because I’m just too tired****. People say to me, “Should we go to the movies?”, “Should we go here?”, “Should we do this?”* ***All I want to do is just curl up on the sofa because I’m too tired. “Just leave me alone****. It’s not a good day today.” My social life is non-existent, I suppose.* | *‘Even going to the cinema, you think, “Oh, I don’t want to go there; I’m so tired.”* ***The meaningful thing to me is I think I’ve got a bit of a life now****, whereas before I never had nothing’.* |
|  |  | *‘I was like a social butterfly. I was going here, there, there, everywhere. Would have people over all the time entertaining. It never bothered me. I love doing that.* ***And I feel like it’s gotten to the point where it’s like I can’t entertain what I used to do, and it’s affected [me] socially that way. I feel that exhaustion just takes over your life****, and it’s hard to describe for people to understand it unless you’ve gone through that feeling of exhaustion yourself’.* | *‘Yeah. There’s days… If the weekends are usually busy.* ***Sometimes I just dread it and I'm like, “I don’t even want to go. I'm exhausted****,” or “I can't. I'm not feeling up to it,” because that exhaustion,* ***it just seems always there because you don’t have energy to communicate and socialize with people some days****’.* |
|  |  | *‘I go out with friends and so on.* ***But obviously I can’t go…if we’re at a party, I can’t dance. If it’s somewhere where lots of people are vaping, that affects my lungs****. I’m a bit careful about where I go, which is a pain. But, yes, mostly family things, I manage quite well’.* | *‘If I’m having a particularly tiring day, one of the days where you haven’t got any energy, that obviously impacts what I’m doing... If I was going out for a meal with friends, that probably would be fine. But* ***if they wanted to go bowling or skating or something like that, I would tend to be a watcher rather than a participant’.*** |
| **Difficulty walking**  n=19, 54% | **Difficulty walking**  n=17, 53% | *‘****Walking is extremely painful. Every single step I take hurts.*** *[…] so I’m limited to how far I can walk. But the breathing side of it as well. It’s hard to explain. The pain makes it harder to breathe in a long period of time’.* | *‘****Walking, as I say, is quite difficult****. I’ve had tests done on my walking and very little of my foot touches the ground when I’m walking. Mainly my heel and my big toe. Everything else sort of arches up if I’m walking normally. I walk up on the pad, where you have to cross this pad, and the computer generates the footprint and its layout. Most of my foot wasn’t touching the floor, unfortunately.* ***I’ve had special insoles done by the hospital and special footwear made where it’s up inside. It’s made it a little bit better, but if I do some walking it’s painful still****’.* |
|  |  | *‘It’s the knee joint on the right and also the ankle on the right.* ***I notice the strain there after a certain time. But I think that’s because I just don’t do the right movement sequence now because the leg doesn’t work properly as a whole, so after a certain time, I get pain from the strain.*** *[…] I have now started cycling a little bit, as well as I can, because of course* ***I still have problems with balance and sometimes also when walking, e.g., I sometimes have blackouts when walking****’.* | *‘I think that comes from the different spatial perceptions, or the* ***responses that come from the left and right side, which are unsymmetrical, and because the right leg is weaker, like with tank tracks, the right leg is weaker****, and that means that the left leg, so to speak, almost always, practically always overtakes the right leg’.* |
|  |  | *‘I started with the muscle aches, the leg pains,* ***not being able to walk. I was shuffling like my legs just felt like there’s muscle weakness****, and then they were tapering me down, slowly tapering me down, but every time they would taper me down past 15 [mg of oral GCs] my symptoms would start to flare again’.* | *‘I had a lot of muscle joint pains, weakness in my legs.* ***I could barely walk****’.* |
| **Difficulty with daily or everyday activities**  n=17,^c^ 49% | **Difficulty with daily or everyday activities**  n=20, 63% | *—^c^* | *‘My breathing got so bad, that* ***I was struggling to get dressed..****.* ***Even though I was on the sofa, I had to kind of… that's when it was kind of at the worst point. I had to actually kind of plan to get off the sofa****. I had to kind of sit up halfway, and then kind of prop myself up, and then lift myself up so I was standing…* ***It felt like that was the hardest thing I've ever done****, to kind of lift myself out of a chair and stuff. It's just crazy’.* |
|  |  | *—^c^* | *‘Honestly, I do less, and I make sure I have my albuterol if I leave the house. I tend to be less active. Or I just suck it up.* ***If there’s something I have to get done, I suck it up and I just…you struggle through it****’.* |
|  |  | *—^c^* | *‘Not so much getting the lawn mower out, but once I’ve done one set of lawns, you’d be like, “Oh, need to sit down now,”* ***whereas now I can do both sets of lawns. And I’m absolutely fine and can carry on and probably do another set if I had too as well****. That’s what I’ve noticed the biggest difference was at home doing bits and pieces’.* |
| **Other impacts^e^**  n=22, 63% | **Other impacts^e^**  n=7^d^, 22% | *‘Just* ***from an emotional perspective, this has had a very large effect on my family****. In the meantime, I had to of course switch from working full time to suddenly always being at home, to suddenly not being able to walk like before. Suddenly having to plan everything completely differently, so anything, even if we go on vacation. These have been severe effects for us. So, in principle,* ***from living quite freely to suddenly being at home all of the time. Pretty difficult****’.* | *—^d^* |
|  |  | *‘But by this point, I thought,* ***“Oh my god. Can my life get any worse?”*** *as I’m struggling to breathe. I’m struggling to walk, and I’ve kind of got arthritis as well. I thought, if it got any worse, just kill me now. It was so terrible’.* | *—^d^* |
|  |  | *‘****I also had some depression because I was no longer able to go jogging. That was something that was really important for me before****, to walk in the woods and to go running. I was never able to do that at all. So, I felt very limited’.* | *—^d^* |

^a^Quotes are shown from the same participant at Interview 1 and Interview 2.

^b^Quotes were analysed only for symptoms that were reported by 14 or more participants in Interview 2.

^c^Quotes were analysed only for impacts that were reported by 18 or more participants in Interview 1.

^d^Quotes were analysed only for impacts that were reported by 16 or more participants in Interview 2.

^e^Other impacts (as reported by the participants) included difficulty planning vacations; being careful about what to eat; restlessness; impact on leisure activities or hobbies; feeling stressed, upset, ill-tempered or in a bad mood; difficulty with motor skills; voice loss; increase in medical appointments; difficulty holding a conversation; impaired quality of life; financial impact; missing out on life experiences; falling; and mental exhaustion.

GCs, oral glucocorticoids.

## Supplementary Table 10. Improvements in symptoms and impacts at Interview 2 from Interview 1, and example quotes

| **Symptoms** | | **Participants with improvement at Interview 2, n (%)** | **Participants with worsening at Interview 2, n (%)** | **Example quotes**  ******* *denotes quotes that are from the same participant at Interview 1 and Interview 2* | |
| --- | --- | --- | --- | --- | --- |
| **Interview 1,  number of mentions, % of total participants (n=35)** | **Interview 2,  number of mentions, % of total participants (n=32)** | **Interview 2,  number of mentions, % of total participants (n=32)** | **Interview 2,  number of mentions, % of total participants (n=32)** | **Interview 1** | **Interview 2** |
| **Difficulty breathing or shortness of breath**  n=31, 89% | **Difficulty breathing or shortness of breath**  n=27, 84% | 18 (56) | 1 (3) | *—* | ********“So today I’m actually feeling, I always say that actually, I’m really feeling pretty good. I have much better lung function. In the tests, that was even at 84 percent, even though I think I was at 60 at the beginning. I have to say that I can do a lot more without getting short of breath. […] I can travel in quick steps in cold, moist air without feeling anything in terms of breathing. I’ve significantly reduced my medication. I am currently at 0.25 milligrams of cortisone. […] So talking about the lungs, I have to say I am really doing much, much better than before.”* |
|  |  |  |  | ********“My chest feels a bit clearer. It’s not completely clear, but…I mean, I still have the cough, still have a bit of wheeze, but it’s better than it was before I started. My peak flow, my lung capacity, I only have the little…the small home device to test it, but that’s improved fairly significantly.”* | ********“Well, yes, it’s all changed. Before I could barely… My state of health before we started and just… In the few months or maybe 6 months or so running up to the beginning of the study, my asthma was really bad. I could barely walk 100 meters without getting out of breath. I couldn’t lift a bag of groceries, and I was out of breath going up the stairs to bed, and now I feel normal. I can do those things, and it’s not bothering me.”* |
|  |  |  |  | *—* | *“I can walk better. I can ride my bike, I couldn’t do that for many years. I’m more resilient simply because I’m able to breathe better and because I just don’t have any more pain.”* |
| **Nasal congestion or discharge**  n=29, 83% | **Nasal congestion or discharge**  n=27, 84% | 16 (50) | 2 (6) | *“Otherwise, during the day, it limits the symptoms, from time to time my nose is still slightly blocked. But no longer severely, as it was before, I can definitely breathe relatively normally and actually walk now if I do it from time to time. Every other day or so. It takes time, but in any case it’s more possible again, so more fitness is there.”* | *—* |
|  |  |  |  | *—* | *“Yes, I do [have nasal discharge], but very rarely now since the treatment. […] the stuffy nose now occurs at longer intervals. I haven’t had it for a long time, I think for nine months. Before that I had it all the time, I’ve had operations because everything was tight.”* |
| **Fatigue**  n=28, 80% | **Fatigue**  n=22, 69% | 15 (47) | 3 (9) | *—* | *“In the past, I very often had very severe fatigue, where I was really unable to do anything. It became less and less, and now it’s almost gone. This is a big change.”* |
| **Neuropathy, numbness, or tingling**  n=26, 74% | **Neuropathy, numbness, or tingling**  n=15, 47% | 9 (28) | 1 (3) | *—* | *—* |
| **Coughing**  n=26, 74% | **Coughing**  n=18, 56% | 11 (34) | 0 (0) | ********“I do have a permanent cough. Sometimes, mostly I swallow what comes up but sometimes I have to cough it out. Yeah, so that’s more or less… actually it hasn’t been so bad just lately, which you could attribute to the first injection possibly. But yeah, it’s not a big problem at the moment but sometimes it’s a really deep-seated cough.”* | ********“They’ve become very mild. I used to have a really bad cough and now, as I said to the doctor, it’s not as deep seated. It’s more superficial and I’m not coughing anywhere near as often, and I don’t have to use the salbutamol inhaler, the fast relief one, at all.”* |
|  |  |  |  | ********“I’m not coughing. Usually when I wake up, I have sort of…within the first 10 or 15 minutes of…it also depends on if I have to get out of bed or not, but I usually…I’m trying to clear my chest. That has changed. I’m not coughing as much. My chest seems clearer anyway.”* | ********“Well, like I said, the improvement in my hearing, the improvement in my breathing, my chest, and I’ve been able to cut down on the steroids, and my cough is gone.”* |
| **Wheezing**  n=24, 69% | **Wheezing**  n=10,^a^ 31% | 10 (31) | 0 (0) | — | —^a^ |
| **Weakness**  n=23, 66% | **Weakness**  n=17, 53% | 10 (31) | 4 (13) | — | — |
| **Skin manifestations**  n=18, 51% | **Skin manifestations**  n=14, 44% | 5 (16) | 4 (13) | — | — |

| **Impact** | | **Participants with improvement at Interview 2, n (%)** | **Participants with worsening at Interview 2, n (%)** | **Example quotes**  ******* *denotes quotes that are from the same participant at Interview 1 and Interview 2* | |
| --- | --- | --- | --- | --- | --- |
| **Interview 1, number of mentions, % of total participants (n=35)** | **Interview 2, number of mentions, % of total participants (n=32)** | **Interview 2, number of mentions, % of total participants (n=32)** | **Interview 2, number of mentions, % of total participants (n=32)** | **First Interview** | **Second Interview** |
| **Ability to exercise or engage in strenuous activities**  n=26, 74% | **Ability to exercise or engage in strenuous activities**  n=25, 78% | 14 (44) | 1 (3) | ********’Since I’ve been in the study, I notice that I’m doing better. Because before, I couldn’t swim 50 meters without hitting the asthma spray again and things like that; now it’s going really great. Now I can once again swim 1,000 meters in 30 minutes, that works really well, it’s great. From that perspective, it’s a huge improvement’.* | ********’I can ride a bike, I can walk, I can even go jogging. That’s much, much, much better’.* |
|  |  |  |  | *‘Yes, it’s a tough one actually. I went out cycling and stuff yesterday. It didn’t feel difficult at all. I think maybe it has helped a bit’.* | *—* |
|  |  |  |  | *—* | *‘No, I’ve had no difficulties, actually. In fact, I’m finding improvement in my running. It’s shuffling. So I’m trying to shuffle quicker than I was before. So, no, it hasn’t impacted that. And I’m working… I’ve not had any negative impact since starting on exercise, no’.* |
| **Quality and quantity of sleep**  n=25, 71% | **Quality and quantity of sleep**  n=26, 81% | 14 (44) | 2 (6) | ********’If I remember rightly, I think I had one very, very, very good night’s sleep, and that was lovely’.* | ********’Yeah, yeah, that has got better. I’m not the world’s best sleeper anyway, but yeah, I do seem to have more restful sleep now’.* |
|  |  |  |  | *‘Yeah, I would say it has, yes. Because I used to have completely uninterrupted blissful sleep before. I was going to say I do still have some nights where I’m undisturbed. But there has been an improvement’.* | *—* |
|  |  |  |  | *—* | *‘Definitely, because you wouldn’t think about it at all. But for me it’s always like that, I live in the now and that’s the way it is now and I accept it. I accepted it afterwards, when I was falling asleep at seven in the evening. But that has definitely changed now, the quality has changed a lot and in a very positive way’.* |
|  |  |  |  | *—* | *‘I’m not wheezing in the middle of the night, which never leads to very good sleep. I’m also breathing better out of my nose, which is also better quality of sleep because mouth breathing is never good for sleeping. The fact that my sinuses have been better controlled means that I’m not having to mouth breathe as much overnight’.* |
| **Ability to work**  n=22, 63% | **Ability to work**  n=17, 53% | 10 (31) | 1 (3) | *‘I have more energy, so I feel more productive at work’.* | *—* |
|  |  |  |  | *‘For instance, this evening. I should finish work at 4:00 and tonight I finished at 5:00 because we were busy, and it’s because I wanted to because I had that little bit of energy. I feel like I’ve got a bit of oomph. You’ve got a bit more going in you. That’s the impact. Whereas before I said, “Sorry, I’m going home now. I’m too tired,” tonight I said, “No, I’ll stay and help.”’.* | *—* |
|  |  |  |  | *—* | *‘Now, if I work? When I work, I walk quite fast. I carry small bins with the mail, so I go back and forth in schools, in nurseries, I have to go to the hospital to get… anything administrative, it’s all papers that I have to get. When I have all these papers, I have to go back to the municipal administration, but I have a car. It means that at some point, I walk, I climb the stairs or I take the elevator and I walk a lot, but it’s easy to walk. I try to rest a little. I’m in my van, I sit down, then I drive. I sit down, then I get to school, I go out, I go get the mail, I come out and it’s fine. I can’t complain’.* |
| **Ability to engage in social activities**  n=21, 60% | **Ability to engage in social activities**  n=20, 63% | 12 (38) | 0 (0) | *‘From going to nothing, no social activities because I was just too tired and didn’t want to go anywhere. I used to go to everybody, “You go. Just leave me alone. Just let me go to bed,” because that’s all I wanted. Now I think every other week I’m going to a social activity whereas before I wouldn’t. I’ve been to the pictures. I’ve had a party I’ve been to. Whereas before you wouldn’t have got me there because I was just too tired’.* | *—* |
|  |  |  |  | *‘I’ve made a couple of ventures out with girlfriends; dinner and stuff that I wasn’t able to do, but now I can. I have the energy to do it. I still have fatigue, but it’s part of the disease. I don’t think that’ll ever go away completely’.* | *—* |
|  |  |  |  | *—* | *‘I would say I’m probably more active going to social activities. I’m accepting more invitations. I’m more willing to be a little be adventurous in the kind of activities I’m doing. I haven’t been as sick this past year, which means that I can definitely go out more often, be around more people’.* |
| **Difficulty walking**  n=19, 54% | **Difficulty walking**  n=17, 53% | 7 (22) | 1 (3) | *—* | *—* |
| **Difficulty with daily or everyday activities**  n=17,^b^ 49% | **Difficulty with daily or everyday activities**  n=20, 63% | 8 (25) | 0 (0) | *—* | *—* |
| **Other impacts^c^**  n=22, 63% | **Other impacts^c^**  n=7, 22% | 2 (6) | 2 (6) | ********’So, with regard to severity, I cannot say right now at all. I’m of course through nearly all of it, because I am fitter overall. Since then, I have not had another fall. Well, I’ve been someone, who also just through this hemiparesis, I fell frequently, but because of this, because of my general condition, overall, I have the feeling that I am better, I also haven’t had any falls anymore’.* | ********’For the leisure activities, the difference is that I can ride my bike again and so on, with, so without, what’s not noticeable to my husband is that I can maintain balance better, that I can put more strain on myself’.* |
|  |  |  |  | *‘Yes, activities in my free time, going into the garden, walking, I’ve done those things. That has always been tiring, more or less, but I did it nevertheless. I hope this will now get even easier’.* | *—* |

^a^Quotes were analysed only for symptoms that were reported by 14 or more participants in Interview 2.

^b^Quotes were analysed only for symptoms that were reported by 18 or more participants in Interview 1.

^c^Other impacts (as reported by the participants) included difficulty planning vacations; being careful about what to eat; restlessness; impact on leisure activities or hobbies; feeling stressed, upset, ill-tempered or in a bad mood; difficulty with motor skills; voice loss; increase in medical appointments; difficulty holding a conversation; impaired quality of life; financial impact; missing out on life experiences; falling; and mental exhaustion.

## Supplementary Table 11. Primary and secondary outcomes

| **Endpoints** |  | **Sub-study**  **Total participants, N=38** | **Main trial**  **Total participants, N=140** |
| --- | --- | --- | --- |
| **Primary endpoint: Main remission at both weeks 36 and 48, n (%)** | BVAS=0 and  Oral GC ≤ 4 mg/day | 24 (63.2) | 80 (57.1) |
|  | Oral GC ≤ 4mg/day | 25 (65.8) | 83 (59.3) |
|  | BVAS=0 | 34 (89.5) | 117 (83.6) |
| **Secondary endpoint: Accrued duration of main remission, n (%)** | 0 week | 4 (10.5) | 24 (17.1) |
|  | 0 to < 12 week | 9 (23.7) | 23 (16.4) |
|  | 12 to < 24 week | 2 (5.3) | 16 (11.4) |
|  | 24 to < 36 week | 11 (28.9) | 39 (27.9) |
|  | ≥ 36 week | 12 (31.6) | 38 (27.1) |
| **Secondary endpoint: Mean daily dose of oral GC during weeks 48 through 52, n (%)** | 0 mg | 13 (34.2) | 48 (34.3) |
|  | > 0 to ≤ 4.0 mg | 19 (50.0) | 49 (35.0) |
|  | > 4.0 to ≤ 7.5 mg | 4 (10.5) | 28 (20.0) |
|  | > 7.5 mg | 2 (5.3) | 15 (10.7) |
| **Secondary endpoint: Reduction in oral GC dose during weeks 48 through 52, n (%)** | ≥ 50% reduction | 31 (81.6) | 111 (79.3) |
|  | 100% reduction | 13 (34.2) | 47 (33.6) |

BVAS, Birmingham Vasculitis Activity Score; GC, glucocorticoid


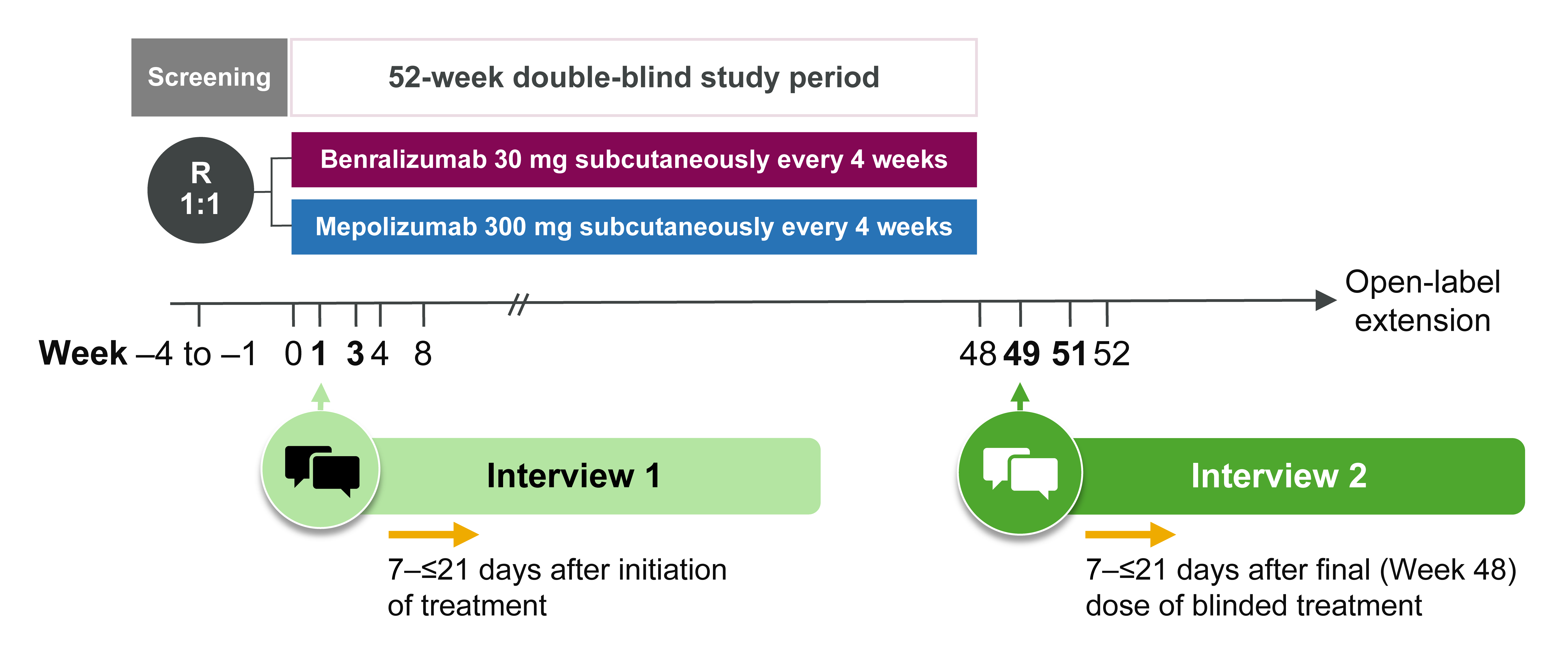


## Supplementary Figure 1. Design of the qualitative sub-study of MANDARA. R, randomisation.


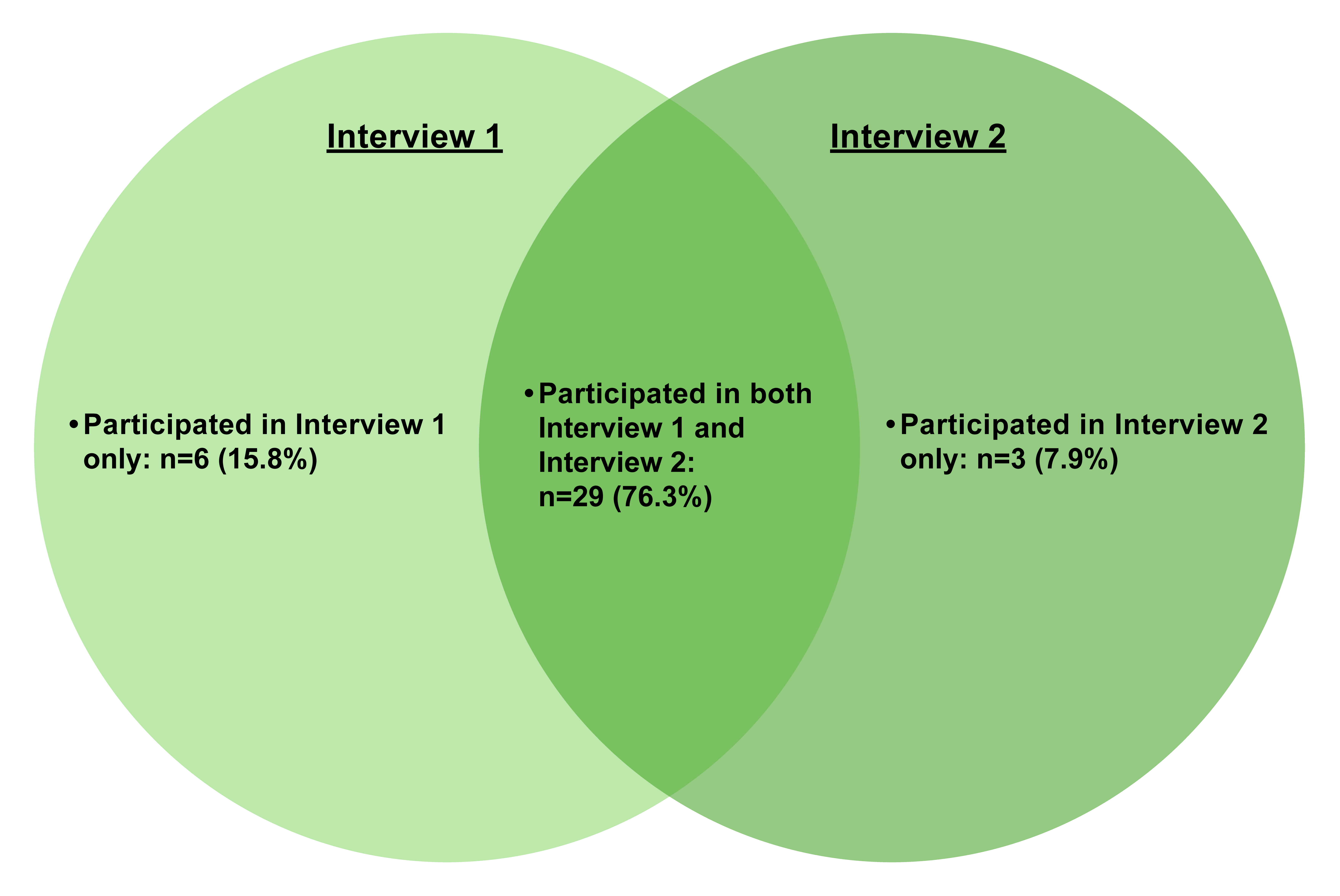


## Supplementary Figure 2. Number of participants in each interview.

Participated in at least one interview: N=38 (100%).

## Reference

1. Maruish ME, Editor. User’s Manual for the SF-36v2 Health Survey. Lincoln (RI): Quality Metric Incorporated; 2011.
